# Complex genetic architecture underlies maize tassel domestication

**DOI:** 10.1111/nph.14400

**Published:** 2017-01-09

**Authors:** Guanghui Xu, Xufeng Wang, Cheng Huang, Dingyi Xu, Dan Li, Jinge Tian, Qiuyue Chen, Chenglong Wang, Yameng Liang, Yaoyao Wu, Xiaohong Yang, Feng Tian

**Affiliations:** ^1^ National Maize Improvement CenterChina Agricultural UniversityBeijing100193China

**Keywords:** candidate gene, genetic architecture, maize, quantitative trait locus (QTL), tassel

## Abstract

- Maize (*Zea mays*) tassels underwent profound morphological changes during maize domestication and improvement. Although a number of genes affecting maize inflorescence development have been identified, the genetic basis of the morphological changes in maize tassels since domestication is not well understood.
- Here, using a large population of 866 maize‐teosinte BC_2_S_3_ recombinant inbred lines genotyped using 19 838 single nucleotide polymorphism (SNP) markers, we performed high‐resolution quantitative trait locus (QTL) mapping for five tassel morphological traits.
- We showed that the five tassel traits were associated with different genetic architecture features. Known genes for maize inflorescence development identified by mutagenesis were significantly enriched in the tassel trait QTLs, and many of these genes, including *ramosa1* (*ra1*), *barren inflorescence2* (*bif2*), *unbranched2* (*ub2*), *zea floricaula leafy2* (*zfl2*) and *barren stalk fastigiate1* (*baf1*), showed evidence of selection. An in‐depth nucleotide diversity analysis at the *bif2* locus identified strong selection signatures in the 5′‐regulatory region. We also found that several known flowering time genes co‐localized with tassel trait QTLs. A further association analysis indicated that the maize photoperiod gene *ZmCCT* was significantly associated with tassel size variation. Using near‐isogenic lines, we narrowed down a major‐effect QTL for tassel length, *qTL9‐1*, to a 513‐kb physical region.
- These results provide important insights into the genetic architecture that controls maize tassel evolution.

## Introduction

**Figure 1:**
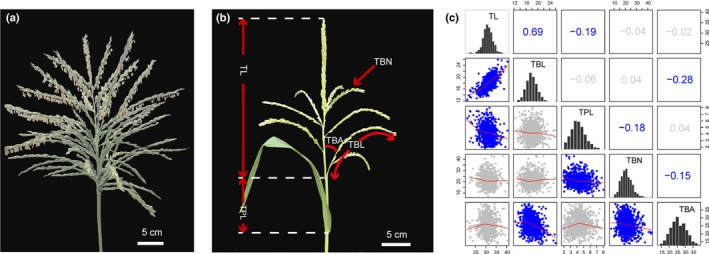
Tassel architecture trait phenotyping and correlation analysis in the maize‐teosinte BC_2_S_3_ recombinant inbred line (RIL) population. (a) The tassel of a typical *Zea mays* ssp. *parviglumis* accession (PI 566686). (b) Tassel architecture traits scored in the BC_2_S_3_ RIL population. (c) Phenotypic analysis. The histograms on the diagonal show the phenotypic distribution of each trait. The plots below the diagonal are scatter plots of the compared traits, and the values above the diagonal are the pairwise correlation coefficients between the traits. Significant phenotypic correlations (*P *<* *0.05) are shown in blue, and insignificant correlations are shown in gray. TL, tassel length; TBN, tassel branch number; TBA, tassel branch angle; TBL, tassel branch length; TPL, tassel peduncle length.

Maize (*Zea mays* ssp. *mays*) was domesticated from its wild ancestor Balsas teosinte (*Zea mays* ssp. *parviglumis*) *c*. 9000 yr ago in southwestern Mexico (Matsuoka *et al*., 2002). Maize and teosinte differ dramatically in a number of traits (Doebley *et al*., 1990; Doebley & Stec, 1991). The tassel is the male inflorescence that terminates the main culm in both maize and teosinte, and it has long been employed as a crucial morphological structure for taxonomical comparisons between maize and teosinte (Doebley, 1983). The tassel morphology of maize has changed significantly since domestication. The most evident difference between maize and teosinte in tassel morphology is that the tassels of *Z. mays* ssp. *parviglumis* are highly branched and usually possess > 50 tassel branches, whereas the tassels of cultivated maize typically possess fewer tassel branches (Fig. 1a). During the past 80 yr of maize improvement, one of the pronounced phenotype changes has been the consistently decreasing tassel size over time (Duvick, 2005). The relationship between tassel size and maize grain yield was first investigated in the early 1890s. Watson (1892) found that the grain yield of detasseled plants was 50.6% higher than that of plants with intact tassels. Subsequent experiments further showed that small tassels are often associated with improvements in grain production efficiency, especially at a high plant density (Grogan, 1956; Duncan *et al*., 1967; Hunter *et al*., 1969), which is because smaller tassels can release more energy for grain production and reduce light interception (Grogan, 1956; Duncan *et al*., 1967; Hunter *et al*., 1969). Therefore, dissecting the genetic mechanisms of tassel variation will help to elucidate the maize domestication and improvement process and better enable directed manipulations of tassel architecture to improve maize grain yield potential.

Mutant and development studies have led to considerable progress in our understanding of maize tassel development (Bommert *et al*., 2005b; Thompson & Hake, 2009; Tanaka *et al*., 2013). Tassel inflorescences are formed from several types of meristem in a strictly organized manner. As plants transition from the vegetative to the reproductive stage, the shoot apical meristem (SAM) converts into the inflorescence meristem (IM), which first initiates several lateral branch meristems (BMs) that become long branches and then produce spikelet pair meristems (SPMs). Each SPM then produces a pair of spikelet meristems (SMs), which in turn produce a pair of floret meristems (FMs). The FMs eventually produce the florets. A number of genes that affect the differentiation steps from IM to FM have been identified (Bommert *et al*., 2005b; Thompson & Hake, 2009; Tanaka *et al*., 2013). For example, *thick tassel dwarf1* (*td1*) and *fasciated ear2* (*fea2*), which encode orthologs of the Arabidopsis *CLAVATA1* (*CLV1*) and *CLV2*, respectively, affect the transition from SAM to IM, and their mutations cause more tassel branches and an overabundance of spikelets on the tassel (Taguchi‐Shiobara *et al*., 2001; Bommert *et al*., 2005a).

The gene *barren inflorescence2* (*bif2*), a maize ortholog of the Arabidopsis serine/threonine kinase *PINOID*, affects the formation of SPM and BM and can decrease the number of branches, spikelets, and florets on the tassel (McSteen & Hake, 2001). *BARREN STALK1* (*BA1*) encodes a basic helix–loop–helix (bHLH) transcription factor and plays a similar role to *bif2* in controlling the initiation and maintenance of the axillary meristems, and the tassels of *ba1* mutants are usually unbranched and shortened (Gallavotti *et al*., 2004). The genes in the *ramosa* pathway, including *ra1*,* ra2* and *ra3*, which encode a C_2_H_2_ zinc‐finger domain‐containing transcription factor, a LATERAL ORGAN BOUNDARY (LOB) domain‐containing transcription factor and trehalose‐6‐phosphate phosphatase (TPP), respectively, play crucial roles in regulating inflorescence branching (Vollbrecht *et al*., 2005; Bortiri *et al*., 2006; Satoh‐Nagasawa *et al*., 2006). Both *indeterminate spikelet1* (*ids1*) and *sister of indeterminate spikelet1* (*sid1*) encode the ERF family of APETALA2 (AP2) transcription factors and act redundantly to control the timely conversion of the SM into the FM and the determinacy of the SM (Chuck *et al*., 1998, 2008). The SQUAMOSA PROMOTER BINDING (SBP)‐box transcription factors *tasselsheath4* (*tsh4*), *unbranched2* (*ub2*) and *unbranched3* (*ub3*) function as redundant factors that regulate the differentiation of lateral primordias (Chuck *et al*., 2010, 2014). The single, double and triple mutants of *tsh4*,* ub2* and *ub3* show a significant reduction in tassel branch number (Chuck *et al*., 2014). Despite the significant progress in cloning these tassel development genes, the extent to which these genes play roles in regulating tassel natural variation is not well understood.

Maize exhibits wide natural variation in tassel morphology. Compared with the large number of quantitative trait locus (QTL) mapping studies for ear architecture, relatively limited information has been published for the QTLs that control tassel architecture. Using an Illinois High Oil × Illinois Low Oil F_1_‐derived S_1_ population, Berke & Rocheford (1999) mapped 16 QTLs for tassel branch angle (TBA), tassel branch number (TBN) and tassel weight (TW). Mickelson *et al*. (2002) reported nine QTLs for TBA and TBN in a B73 × Mo17 recombinant inbred population. Upadyayula *et al*. (2006) conducted QTL mapping for a comprehensive set of tassel architecture traits in a BC_1_S_1_ population and detected QTLs that regulate different steps of inflorescence development. Small population size and limited marker density prevent an accurate estimation of the genetic architecture of maize tassel natural variation and the identification of the underlying genes. Using a maize nested association mapping (NAM) population that consists of 25 recombinant inbred line (RIL) populations, each containing 200 RILs (McMullen *et al*., 2009), Brown *et al*. (2011) conducted large‐scale QTL mapping and genome‐wide association mapping for four tassel traits. This study identified 33–39 QTLs and 241–325 single nucleotide polymorphism (SNP) associations for the four tassel traits, and the results showed that distinct genetic architectures underlie maize ear and tassel natural variation. Recently, Wu *et al*. (2016) used nearly 8000 inbred lines, including two sets of NAM populations (US NAM and CN NAM), and a global association panel to dissect the genetic architecture of two tassel traits, TBN and tassel length. A total of 125 QTLs and 965 quantitative trait nucleotides (QTNs) were identified for the two tassel traits. In addition to detecting the known genes in the QTL regions, Wu *et al*.'s study also identified several new candidate genes. These genetic mapping studies have provided abundant insights into the genetic factors that influence tassel variation within maize populations; however, comprehensive genetic mapping studies have not been performed to identify the genetic architecture that controls the morphological differences in the tassels between maize and its wild ancestor, teosinte.

In this study, a large population of 866 maize‐teosinte BC_2_S_3_ RILs that were genotyped using 19 838 SNP markers was used to perform high‐resolution QTL mapping for five tassel architecture traits, including tassel length, tassel branch length, tassel peduncle length, TBN, and TBA. We characterized the genetic architecture of these tassel traits and evaluated the contribution of known inflorescence development genes in regulating tassel natural variation. Using association mapping, we showed that the maize photoperiod gene *ZmCCT* may play an important role in regulating maize tassel evolution. Finally, using near‐isogenic lines (NILs) developed from a heterogeneous inbred family (HIF), we narrowed down a major‐effect QTL for tassel length, *qTL9‐1*, to a 513‐kb physical region.

## Materials and Methods

### Materials

A large population of 866 BC_2_S_3_ RILs derived from a cross between W22, a typical temperate maize (*Zea mays* ssp. *mays*) inbred line and CIMMYT accession 8759, a typical accession of teosinte (*Zea mays* ssp. *parviglumis*) was obtained from the Maize Coop Stock Center. Detailed information on the BC_2_S_3_ RIL population has been previously reported (Shannon, 2012; Huang *et al*., 2016; Li *et al*., 2016). The 866 maize‐teosinte BC_2_S_3_ RILs were previously genotyped using 19 838 SNPs (Shannon, 2012). As a consequence of the large sample size and high‐density markers, this population has been used as a powerful tool for trait dissection and gene cloning (Hung *et al*., 2012; Lin *et al*., 2012; Wills *et al*., 2013; Lang *et al*., 2014; Huang *et al*., 2016; Li *et al*., 2016).

### Field trials and phenotypic analysis

The maize‐teosinte BC_2_S_3_ RILs were planted at the Shangzhuang Experimental Station of China Agricultural University, Beijing (39.9°N, 116.4°E), China, in the summers of 2012 and 2013. The field trials were the same as those described by Li *et al*. (2016), who measured leaf number and flowering time in the population. Briefly, in each trial, the 866 BC_2_S_3_ RILs were grown according to an augmented incomplete randomized block design that has been widely used to phenotype large populations (Buckler *et al*., 2009; Kump *et al*., 2011; Tian *et al*., 2011; Huang *et al*., 2016; Li *et al*., 2016). Two maize inbred lines, W22 and Mo17, were randomly inserted in each incomplete block as controls. Each line was grown in a three‐row plot in 2012 and a single‐row plot in 2013, with 15 plants per row, 25 cm between plants within each row and 50 cm between rows.

Five tassel architecture traits, including tassel length (TL), TBN, tassel branch length (TBL), tassel peduncle length (TPL) and TBA (Fig. 1b), were measured 15 d after anthesis in the BC_2_S_3_ RIL population. TL is the length from the lowermost primary branch to the tip of the tassel (cm); TBN is the total number of tassel branches, including the primary and secondary branches; TBL is the length of the lowermost primary branch (cm); TPL is the length from the ligule region of the flag leaf to the lowermost primary branch node (cm); and TBA is the angle between the inflorescence main stem and the lowermost primary branch.

Following a previously described approach that minimizes the effects of environmental variation (Buckler *et al*., 2009; Kump *et al*., 2011; Tian *et al*., 2011; Huang *et al*., 2016; Li *et al*., 2016), the phenotypic data collected over 2 yr were fitted with a linear mixed model that included effects for the year, the genotype, incomplete blocks, the field range and the field row. For each line, the best linear unbiased predictor (BLUP) was predicted with Sas (v.9.2; SAS Institute Inc., Cary, NC, USA) and used as the phenotypic input in subsequent QTL mapping. Based on the linear mixed model and following the previously described method (Holland *et al*., 2003; Coles *et al*., 2010), the line mean‐based broad‐sense heritability for each trait was estimated as $H^{2}=\sigma_{g}^{2}/\left(\sigma_{g}^{2}+\sigma_{e}^{2}/n\right)$, where $\sigma_{g}^{2}$ is the genetic variance, $\sigma_{e}^{2}$ is the residual error variance, and *n* is the number of years.

### QTL mapping

The QTL mapping method has been previously described (Shannon, 2012; Huang *et al*., 2016; Li *et al*., 2016). Briefly, a modified version of R/qtl software that considers the BC_2_S_3_ pedigree of the RILs (Broman *et al*., 2003) was used for QTL mapping (Shannon, 2012). A multiple QTL mapping procedure was used to identify the QTLs. For each trait, a total of 1000 permutation tests were used to determine the *P *<* *0.05 logarithm of odds (LOD) significance threshold level for claiming QTLs. The *scanone* command, which uses simple interval mapping, was first used to identify an initial QTL list for subsequent multiple QTL fitting (Broman *et al*., 2003). The multiple QTL model was confirmed using a drop‐one ANOVA, and only the QTLs with an LOD score greater than the threshold and an ANOVA *P*‐value < 0.05 were retained in the model. The *refineqtl* command was then used to further refine the position of each QTL in the model. The likelihood ratio test was used to measure the improvement of the model. Finally, the *addqtl* tool was used to search for additional QTLs. The ANOVA and *refineqtl* procedures were repeated to determine whether the newly added QTLs could improve the model. The entire process was repeated until significant QTLs could no longer be added. The total phenotypic variation explained by all QTLs was calculated from a full model that fitted all QTL terms in the model using the *fitqtl* function. The percentage of phenotypic variation explained by each QTL was estimated using a drop‐one‐ANOVA analysis implemented with the *fitqtl* function. The confidence interval for each QTL was defined using a 2‐LOD support interval.

### Co‐localization with inflorescence development genes

We compiled a gene list that contains 31 known inflorescence development genes obtained from previous publications (Supporting Information Tables S1, S2). The number of inflorescence development genes that are located in the support interval of tassel QTLs was counted. To determine whether the observed number of co‐localizations was significant, 1000 permutations were performed to evaluate the likelihood of the co‐localization between the known inflorescence development genes and the tassel QTLs occurring by chance alone. To control the influence of QTLs with large support intervals, QTLs with a support interval > 20 Mb were excluded from the co‐localization analysis; thus, 61 QTLs were retained for the subsequent analysis. Because of the overlapping support intervals of many QTLs, the 61 QTLs were further merged into 47 nonoverlapping regions. None of the 47 merged regions were > 20 Mb. For each permutation, 47 nonoverlapping regions were randomly assigned to the maize genome, and the number of known inflorescence development genes residing in the simulated QTL regions was counted. The 1000 permutations produced a simulated distribution of the number of known inflorescence genes located in the randomly generated nonoverlapping regions. With this null distribution, we determined the probability of obtaining the observed number of co‐localizations at *P *<* *0.05.

### QTL effect validation and fine mapping

To validate the phenotypic effects of the *ra1* locus, NILs were developed from an HIF that is heterozygous only in the *ra1* region (Fig. S1a). Within a small HIF‐derived F_2_ family, plants that were homozygous for the W22 allele and teosinte allele (designated NIL^maize^ and NIL^teosinte^, respectively) were identified using markers across the region and then selfed for progeny phenotype testing. NIL^maize^ and NIL^teosinte^ were planted in neighboring rows and scored for the tassel traits TL, TBN, and TBL as well as other agronomic traits, including plant height, ear height, leaf length, leaf width and days to anthesis. A *t*‐test was used to determine the significance of phenotypic differences between NIL^maize^ and NIL^teosinte^ at *P *<* *0.01.

To perform fine mapping of *qTL9‐1*, a large F_2_ population derived from an HIF that was heterozygous only at *qTL9‐1* (Fig. S1b) was planted in a winter nursery in Hainan Province in 2013. Recombinants were identified from the F_2_ population using markers across the target region. A similar within‐family comparison strategy (Hung *et al*., 2012; Huang *et al*., 2016; Li *et al*., 2016) was used to delimit the region of *qTL9‐1*. Briefly, within each recombinant‐derived F_3_ family, homozygous recombinant (HR) and homozygous nonrecombinant (HNR) plants were identified using appropriate markers. The significance of phenotypic differences between the HR and HNR plants within each family was determined. If a significant phenotypic difference was observed between the HR and HNR plants, the parental F_2_ recombinant was heterozygous for the target QTL; otherwise, the recombinant was homozygous for either parent. To evaluate the significance of the phenotypic differences between the HR and HNR plants (*P *<* *0.01), *t*‐tests with Bonferroni corrections for multiple testing were used. A substitution mapping procedure (Paterson *et al*., 1990) was used to delimit the causal QTL region. For each recombinant, the source of the QTL effect was first determined by comparing the marker genotypes of the HR and HNR plants. Through integrating the QTL location information from all recombinants, the causal QTL region can be delimited in a stepwise manner to a marker interval.

### Molecular analyses

DNA was extracted from fresh leaves using the CTAB method (Murray & Thompson, 1980) with minor modifications. To develop polymorphic markers, specific primers were developed based on the B73 reference genome (B73 RefGen_v2) (http://www.maizegdb.org/) using the software Primer 3.0 (http://bioinfo.ut.ee/primer3-0.4.0/). PCR was performed as described previously (Huang *et al*., 2016), and PCR products were directly sequenced in both directions. Sequence alignments were performed with bioedit (v.7.0.9.0; North Carolina State University, Raleigh, NC, USA) and manually edited if necessary. Sequences that showed large insertions or deletions were further developed into InDel markers. If there was no large insertion or deletion difference between the sequences of parents, restriction analysis was conducted to develop cleaved amplified polymorphic sequence (CAP) markers.

### DNA sequencing and selection test

The nucleotide diversity around the *bif2* region was investigated in 28 diverse maize inbred lines that maximize the genetic diversity of maize (Liu *et al*., 2003; Yu *et al*., 2008) and 21 teosinte accessions that represent a wide geographical distribution of wild teosinte (www.panzea.org). Information on these materials is provided in Table S3. A total of seven pairs of primers were used to amplify a 4.4‐kb sequence around *bif2* (accession numbers KY229920–KY230160). Sequencing reactions were performed on the PCR products in both directions. Multiple sequence alignments were performed using bioedit and manually edited if necessary. The number of segregating sites (S), nucleotide polymorphisms (θ) (Watterson, 1975), nucleotide diversity (π) (Tajima, 1983), and Tajima's *D* statistic (Tajima, 1989) were estimated using dnasp v.5.10.00 (Librado & Rozas, 2009). Insertions and deletions were not included in the analysis. The retention of nucleotide diversity, which is the relative ratio of π in maize to π in teosinte, was calculated for each sequenced region. We evaluated whether the observed loss of genetic diversity in maize relative to that in teosinte could be explained by a domestication bottleneck alone. Following a previously described procedure (Tian *et al*., 2009), coalescent simulations that incorporated the domestication bottleneck (Eyre‐Walker *et al*., 1998; Tenaillon *et al*., 2004) were performed for each region using the ms program (Hudson, 2002). All parameters in the model were assigned to previously established values (Wright *et al*., 2005; Tian *et al*., 2009). The severity of the maize domestication bottleneck (*k*), which is the ratio of the population size during the bottleneck (*N*_b_) to the duration of the bottleneck (*d*), was 2.45 (Wright *et al*., 2005; Tian *et al*., 2009). The population mutation and population recombination parameters were estimated from the teosinte data. For each region, a total of 10 000 coalescent simulations were performed. Significant deviations from the expectations under a neutral domestication bottleneck indicate that selection might have strongly shaped the genetic diversity of the examined region.

## Results

### Large phenotypic variation in tassel architecture traits

Five tassel architecture traits, including TL, TBL, TPL, TBN and TBA (Fig. 1b), were measured in the 866 maize‐teosinte BC_2_S_3_ RIL population. As shown in Fig. 1(c), the maize‐teosinte BC_2_S_3_ RIL population exhibited wide phenotypic variation in the five tassel architecture traits. All tassel traits showed continuous and approximately normal distributions, indicating a quantitative genetic control of the traits studied. TL and TBL were highly correlated, with a correlation coefficient of 0.69; TBL and TBA were moderately correlated, with a correlation coefficient of −0.28; whereas the correlations between TL and TPL, TPL and TBN, TBA and TBN were relatively weak, with absolute values of correlation coefficients of < 0.2.

### Distinct genetic architectures underlie tassel traits

**Figure 2:**
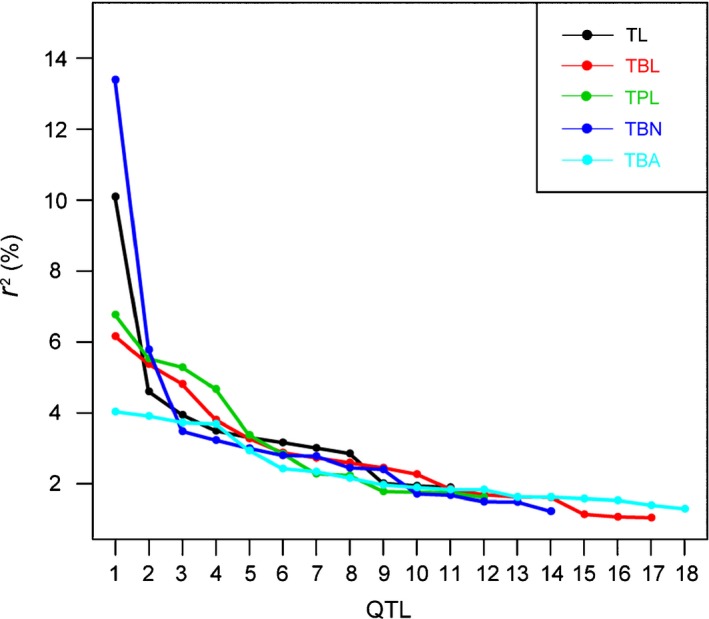
Distribution of phenotypic variation explained by each quantitative trait locus (QTL). QTLs for each trait were ordered according to the proportion of phenotypic variance explained by the QTL (*r* ^2^). TL, tassel length; TBL, tassel branch length; TPL, tassel peduncle length; TBN, tassel branch number; TBA, tassel branch angle.

A total of 72 QTLs were detected for the five tassel traits (Tables 1, S4). For each trait, 11–18 QTLs were detected, and they jointly explained 35.8–55.6% of the total phenotypic variation (Tables 1, S4). According to the distribution of the proportion of phenotypic variance explained by each QTL (*r* ^2^), the five tassel traits are controlled by two contrasting genetic architectures (Fig. 2).

**Table 1:** Summary of the quantitative trait loci (QTLs) identified for five tassel architecture traits in the maize‐teosinte BC_2_S_3_ population

Traits	*H* ^2^ (%)^a^	No. of QTLs	Variation explained by each QTL (%)	Variation explained by all QTLs (%)	Genetic architecture feature
TL	68.2	11	1.9–10.1	43.6	A large‐effect QTL plus many small‐effect QTLs
TBL	71.1	17	1.0–6.2	55.6	Many small‐effect QTLs
TPL	62.8	12	1.6–6.8	35.8	Many small‐effect QTLs
TBN	na	14	1.2–13.4	49.6	A large‐effect QTL plus many small‐effect QTLs
TBA	67.8	18	1.3–4.0	48.3	Many small‐effect QTLs

a Broad‐sense heritability estimates for the tassel traits. Tassel branch number (TBN) was only measured in 2012, and thus the broad‐sense heritability could not be calculated (not available(na)). TL, tassel length; TBL, tassel branch length; TPL, tassel peduncle length; TBA, tassel branch angle.

The TL and TBN traits were found to be controlled by a single large‐effect QTL (*r* ^2^ > 10%) plus many small‐effect QTLs (Fig. 2). Of the detected QTLs for TL and TBN, the QTL with the largest effect showed a more than two‐fold greater effect than the QTL with the second‐largest effect (Fig. 2). In particular, *qTL9‐1* on chromosome 9 was a major‐effect QTL for tassel length, and the maize allele at this QTL could decrease tassel length by 1.3 cm (Table S4). *qTBN7‐1* was a major‐effect QTL for TBN, and the teosinte allele at this locus could increase the number of tassel branches by 3.3 (Table S4).

The other three tassel traits TBL, TPL and TBA were characterized by many small‐effect QTLs (Fig. 2; Table 1). The phenotypic variation explained by each QTL ranged from 1.0% to 6.8%. The effect sizes of the largest‐effect and second‐largest‐effect QTLs were of similar magnitude (Fig. 2).

### Inflorescence development genes are significantly enriched in tassel QTLs

**Figure 3:**
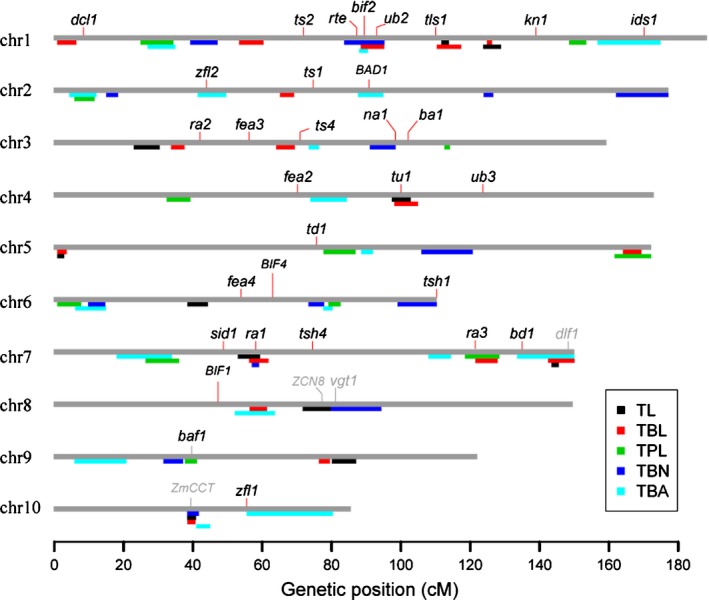
Genomic distribution of tassel trait quantitative trait loci (QTLs) and known inflorescence development genes. The 10 maize chromosomes are indicated by gray bars. Black, red, green, blue and light blue boxes below the chromosomes indicate the 2‐LOD support intervals of the QTLs for tassel length (TL), tassel branch length (TBL), tassel peduncle length (TPL), tassel branch number (TBN) and tassel branch angle (TBA), respectively. *dcl1, dicer‐like 1*;* ts2, tassel seed2*;* rte, rotten ear; bif2, barren inflorescence2*;* ub2, unbranched2*;* tls1, tassel‐less1*;* kn1, knotted1*;* ids1, indeterminate spikelet1*;* zfl2, zea floricaula leafy2*;* ts1, tassel seed1*;* BAD1, BRANCH ANGLE DEFECTIVE 1*;* ra2, ramosa2*;* fea3, fasciated ear3*;* ts4, tasselseed4*;* na1, nana plant1*;* ba1, barren stalk1*;* fea2, fasciated ear2*;* tu1, tunicate1*;* ub3, unbranched3*;* td1, thick tassel dwarf1*;* fea4, fasciated ear4*;* BIF4, BARREN INFLORESCENCE4*;* tsh1, tasselsheath1*;* sid1, sister of indeterminate spikelet1*;* ra1, ramosa1*;* tsh4, tasselsheath4*;* ra3, ramosa3*;* bd1, branched silkless1*;* dlf1, delayed flowering1*;* BIF1, BARREN INFLORESCENCE1*;* ZCN8, Zea mays CENTRORADIALIS 8*;* vgt1, vegetative to generative transition 1*;* baf1, barren stalk fastigiate1*;* zfl1, zea floricaula/leafy1*. The black tick marks above the chromosome indicate the known inflorescence development genes, and the gray ones indicate known flowering time genes.

In previous studies, a number of genes that control maize inflorescence development have been cloned through mutant analysis (Bommert *et al*., 2005b; Thompson & Hake, 2009; Tanaka *et al*., 2013). The mutations at these genes are usually associated with severe defects in tassel morphology; therefore, we determined whether these inflorescence development genes co‐localized with tassel QTLs. Out of 31 known inflorescence development genes (Tables S1, S2), 13 genes were located in the 2‐LOD support intervals of the QTLs for the five tassel traits (Table 2; Fig. 3), which is unlikely to have occurred by chance alone according to a permutation analysis (*P *<* *0.001; Fig. S2). This result suggests that some inflorescence development genes identified by mutagenesis contribute to regulation of natural variation in tassel traits.

**Table 2:** Inflorescence development genes located within quantitative trait locus (QTL) support intervals

Gene	Gene model	Annotation	In selection features?*	Chr	Pos (bp)	TL	TBL	TPL	TBN	TBA
*rte*	GRMZM2G166159	Boron transporter protein	No	1	149 314 799				√	
*bif2*	GRMZM2G171822	Protein kinase	Yes	1	173 847 628		√		√	√
*ub2*	GRMZM2G160917	SBP‐box family protein	Yes	1	188 183 917		√		√	
*ids1*	GRMZM5G862109	AP2 domain containing protein	No	1	292 891 862					√
*zfl2*	GRMZM2G180190	Transcription factor FL	Yes	2	12 643 301					√
*BAD1*	GRMZM2G110242	TCP family TF	No	2	179 981 280					√
*na1*	GRMZM2G449033	Steroid reductase	No	3	178 992 603				√	
*tu1*	GRMZM2G370777	MADS‐box family protein	No	4	178 890 882	√	√			
*tsh1*	GRMZM2G325850	Zinc finger TF	No	6	166 077 729				√	
*ra1*	GRMZM2G003927	Zinc finger TF	Yes	7	110 331 879	√	√		√	
*ra3*	GRMZM2G014729	Glycosyl hydrolase	No	7	166 858 667			√		
*bd1*	GRMZM2G307119	AP2 domain containing protein	No	7	172 208 465					√
*baf1*	GRMZM2G072274	AT‐hook protein	Yes	9	21 966 311			√		

√, corresponding inflorescence gene resides in the 2‐LOD support interval of a tassel QTL; * indicates whether the genes were included in the selection gene list of Hufford *et al*. (2012); *rte, rotten ear*;* bif2, barren inflorescence2*;* ub2, unbranched2, ids1, indeterminate spikelet1*;* zfl2, zea floricaula leafy2*;* BAD1, BRANCH ANGLE DEFECTIVE 1*;* na1, nana plant1*;* tu1, tunicate1*;* tsh1, tasselsheath1*;* ra1, ramosa1*;* ra3, ramosa3*;* bd1, branched silkless1*;* baf1, barren stalk fastigiate1*; TL, tassel length; TBL, tassel branch length; TPL, tassel peduncle length; TBN, tassel branch number; and TBA, tassel branch angle.

**Figure 4:**
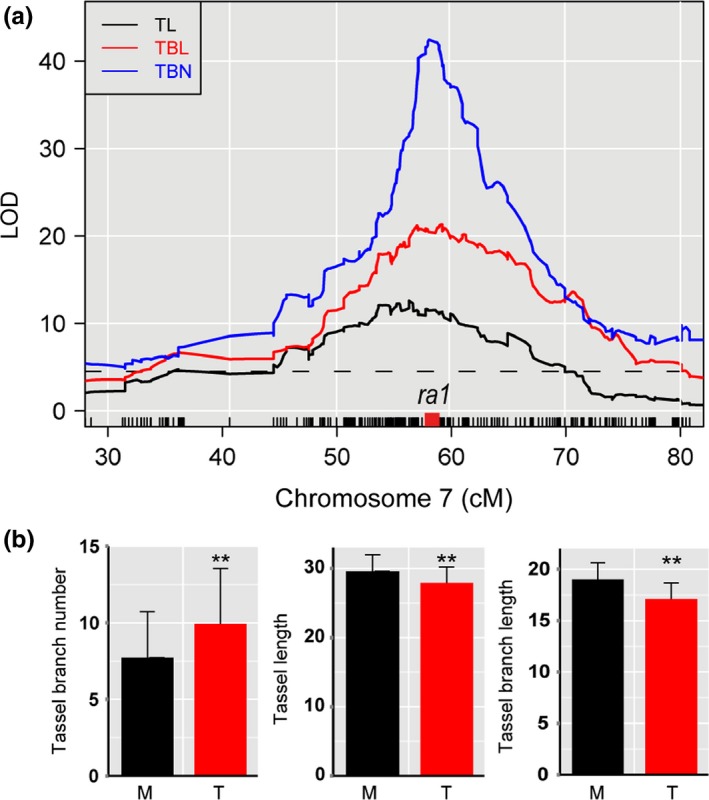
*ramosa1* (*ra1*) co‐localizes with three tassel trait quantitative trait loci (QTLs). (a) LATERAL ORGAN BOUNDARY (LOD) plots of co‐localized tassel trait QTLs (*qTL7‐1*,*qTBN7‐1* and *qTBL7‐1*) at the *ra1* locus. The gray horizontal line represents the *P *<* *0.05 LOD significance threshold as determined by the permutation test. The red square box above the *x*‐axis indicates the position of *ra1*. TL, tassel length; TBL, tassel branch length; TBN, tassel branch number; LOD, logarithm of odds (b) Phenotypic effect analyses of the near‐isogenic lines carrying the maize (*Zea mays* ssp. *mays*) allele (M) and the teosinte (*Zea mays* ssp. *parviglumis*) allele (T) at the *ra1* locus. The phenotypic values are shown as the mean ± SD (*n *>* *50). **, *P *<* *0.01.

Of the 13 inflorescence development genes that co‐localized with the tassel QTLs, the four genes *ra1*,* bif2*,* ub2* and *tunicate1* (*tu1*) resided in the support interval of multiple tassel QTLs, which may indicate their pleiotropy in the control of tassel natural variation. For example, *ra1*, a C_2_H_2_ zinc finger transcription factor that affects maize inflorescence branching (Vollbrecht *et al*., 2005), was near the peak of *qTL7‐1*,* qTBN7‐1* and *qTBL7‐1* (Fig. 4a). At the *ra1* locus, the maize allele was associated with reduced TBN and increased TL and TBL (Table S4). To further validate the phenotypic effects at the *ra1* locus, NILs carrying the W22 allele (NIL^maize^) and the teosinte allele (NIL^teosinte^) at the *ra1* locus were developed from an HIF that segregates only for the *ra1* locus and were scored for tassel traits and other agronomic traits. Compared with NIL^teosinte^, NIL^maize^ showed a significantly decreased TBN and increased TL and TBL (Fig. 4b), which is consistent with the QTL mapping results in the BC_2_S_3_ population. For other agronomic traits, NIL^maize^ and NIL^teosinte^ exhibited significant phenotypic differences in leaf length and days to anthesis, but significant effects were not observed for plant height, ear height, leaf number and leaf width (Table S5).

### Genes showing signals of domestication selection

Because many inflorescence development genes appear to co‐localize with the QTLs that control tassel morphological differences between maize and teosinte, we speculate that these genes might be selection targets during maize domestication and improvement. To address this question, we examined whether the inflorescence development genes that co‐localized with tassel QTLs were included in the selection gene list of Hufford *et al*. (2012), who used a population genomics approach to identify a list of genes that are likely to have been under strong selection during maize domestication and improvement. Interestingly, we found that the five inflorescence development genes *ra1*,* bif2*,* ub2*,* zea floricaula leafy2* (*zfl2*), and *barren stalk fastigiate1* (*baf1*) showed evidence of selection (Table 2). Sigmon & Vollbrecht (2010) performed an in‐depth investigation of the nucleotide diversity around the *ra1* region in a diverse panel of teosintes and maize landraces. They found that the nucleotide diversity in the 5′ and 3′ noncoding regions of *ra1* was significantly reduced in maize landraces compared with teosintes. Further selection tests showed that the 5′ and 3′ noncoding regions are under strong directional selection, indicating that *ra1* is a domestication locus.

### Evidence of selection at the *bif2* locus

**Figure 5:**
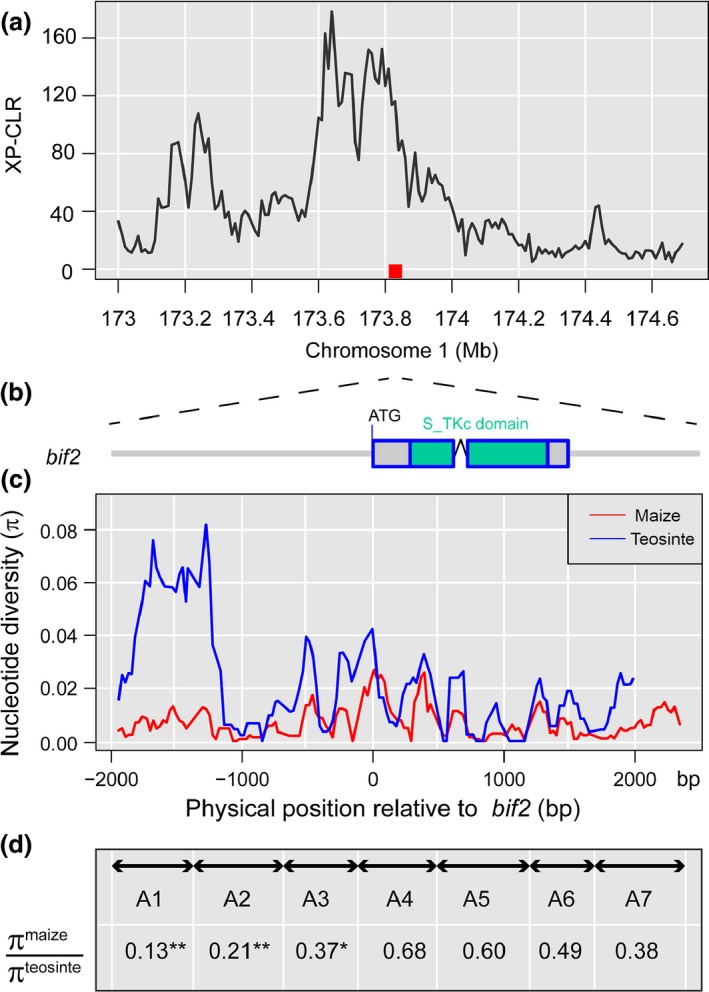
Evidence of selection at *barren inflorescence2* (*bif2*). (a) Cross‐population composite likelihood ratio test (XP‐CLR) plot showing a strong selection signal near *bif2* (Hufford *et al*., 2012). The red square box above the *x*‐axis indicates the position of *bif2*. (b) Sequenced regions at *bif2*. *bif2* is 1489 bp long and consists of two exons. The green box represents the serine/threonine protein kinases catalytic domain. In addition to the coding region, a 2‐kb 5′ noncoding sequence and a 0.9‐kb 3′ noncoding sequence were sequenced in the diverse maize inbred lines and teosinte accessions. (c) Level of nucleotide diversity (π) for maize and teosinte. A sliding window analysis was performed to estimate the nucleotide diversity along the sequenced region. The blue and red curves represent the nucleotide diversity in the maize and teosinte samples, respectively. All positions on the *x*‐axis are shown relative to the start codon of *bif2*. (d) Retention of nucleotide diversity in maize relative to that in teosinte along the sequenced region (measured as the relative ratio of π in maize to π in teosinte). The black arrows indicate the seven amplicons (A1–A7) that were used to sequence the *bif2* region. The values marked with asterisks indicate that significant departures from expectations under a neutral maize domestication bottleneck were detected in the regions (*, *P *<* *0.05; **, *P *<* *0.01).

The gene *bif2* is a maize ortholog of the Arabidopsis *PINOID* gene that controls the initiation and maintenance of axillary meristems (McSteen & Hake, 2001). The *bif2* mutant showed decreased tassel branches and spikelets (McSteen & Hake, 2001). Pressoir *et al*. (2009) performed a candidate gene association analysis at *bif2* and detected significant associations for a variety of tassel traits. In our study, we found that *bif2* resides in the 2‐LOD support intervals of multiple tassel QTLs (Table 2). These results strongly indicate the functional importance of *bif2* in regulating tassel variation. Interestingly, a previous population genomic analysis detected a strong selection signal near *bif2* (Hufford *et al*., 2012) (Fig. 5a), potentially suggesting the role of *bif2* in maize tassel domestication. To further delimit the region affected by selection at the *bif2* locus, we sequenced a 4.4‐kb region, including a 1.5‐kb *bif2* coding sequence, 2‐kb 5′ noncoding sequence and 900‐bp 3′ noncoding sequence, in a panel of diverse maize inbred lines and teosinte accessions using seven amplicons (Tables S3, S6). The nucleotide diversity statistics of each amplicon in the maize and teosinte lines were calculated (Fig. 5c; Table S7).

We examined the amount of nucleotide diversity retained in maize relative to that in teosinte across the sequenced region, which was measured as the relative ratio of π in maize to π in teosinte (Fig. 5d). The retention of genetic diversity significantly varied across the sequenced region. In the coding region of *bif2* (A4, A5 and part of the A6 region), maize retained 49.3–68.3% of the nucleotide diversity found in teosinte, whereas in the 5′ and 3′ noncoding regions, maize retained much less nucleotide diversity compared with teosinte. Particularly, in the A1 region which is 1.4‐kb upstream of *bif2*, maize retained only 13.4% of the nucleotide diversity of teosinte, suggesting possible selection at this region. We used a coalescent simulation that incorporated the maize domestication bottleneck (Eyre‐Walker *et al*., 1998; Tenaillon *et al*., 2004; Wright *et al*., 2005) to estimate the probability of the observed loss of genetic diversity in maize relative to that of teosinte. Following the previously described procedure (Tian *et al*., 2009), we simulated the sequence evolution for each region under the bottleneck model with the parameters established in Wright *et al*. (2005). Significant deviations from expectations under a neutral domestication bottleneck were detected in the A1 (*P *<* *0.01), A2 (*P *<* *0.01) and A3 regions (*P *<* *0.05) (Fig. 5d; Table S7), suggesting that the severe loss of genetic diversity in these regions in maize relative to that in teosinte cannot be explained by the maize domestication bottleneck alone. Thus, our results indicated that the 5′ regulatory region of *bif2* was probably the target of selection during maize domestication.

### Flowering time genes affect maize tassel morphology

**Figure 6:**
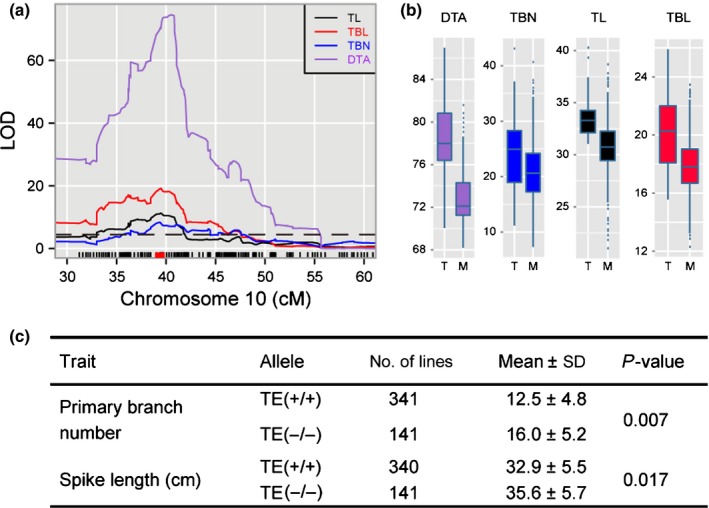
*ZmCCT* affects tassel architecture. (a) LATERAL ORGAN BOUNDARY (LOD) plots of co‐localized tassel trait quantitative trait loci (QTLs) (*qTL10‐1*,*qTBN10‐1* and *qTBL10‐1*) and flowering time QTLs at the *ZmCCT* locus. The gray dashed horizontal line represents the *P *<* *0.05 LOD significance threshold as determined by the permutation test. The red square box above the *x*‐axis indicates the position of *ZmCCT*. LOD, logarithm of odds. (b) Additive effect of the *ZmCCT* locus on tassel and flowering time traits. Phenotypes from the maize‐teosinte BC_2_S_3_ population were classified into teosinte and maize groups according to the peak marker of the corresponding QTL. TL, tassel length; TBL, tassel branch length; TBN, tassel branch number; DTA, days to anthesis. M, NIL ^maize^; T, NIL ^teosinte^. (c) Association analysis of the CACTA‐like transposable element (TE) at the *ZmCCT* promoter with the tassel traits in the association panel. TE (+/+) indicates lines carrying the TE insertion at the *ZmCCT* promoter, and TE (−/−) indicates lines without the TE insertion. The mixed linear model that corrects for both population structure and family relatedness was used to test the association.

In addition to the maize inflorescence development genes, other types of genetic factors might have had an effect on the natural variation in the tassel. We found that several known flowering time genes, including *ZmCCT*,* Zea mays CENTRORADIALIS 8* (*ZCN8*), *delayed flowering1* (*dlf1*) and *vegetative to generative transition 1* (*vgt1*), co‐localized with several tassel trait QTLs (Fig. 3). In particular, *ZmCCT*, which encodes a CCT domain‐containing transcription factor (Hung *et al*., 2012; Yang *et al*., 2013), was close to the QTL peaks of three tassel QTLs (*qTBN10‐1*,* qTL10‐1* and *qTBL10‐1*). The maize allele at the *ZmCCT* locus, which accelerates flowering, was associated with reduced TBN, TL and TBL (Fig. 6b). A previous study showed that a CACTA‐like transposable element (TE) within the *ZmCCT* promoter was the causative variant in reducing photoperiod sensitivity and was the target of a strong selective sweep during the postdomestication spread of maize (Yang *et al*., 2013). To determine whether *ZmCCT* is the underlying gene of the three co‐localized tassel QTLs on chromosome 10, we examined the association of the TE insertion at the promoter of *ZmCCT* with the spike length and primary branch number in an association panel that consisted of 508 diverse maize inbred lines (Yang *et al*., 2013). Using the same mixed linear model that was used to test the association between *ZmCCT* and flowering time in the association panel (Yang *et al*., 2013), we found that the TE at the *ZmCCT* promoter showed significant associations with both the primary branch number and spike length (*P *<* *0.05, Fig. 6c). This result suggests that *ZmCCT* shows pleiotropic effects on flowering time and tassel architecture.

### Fine mapping of *qTL9‐1*

QTL mapping for tassel length detected a major‐effect QTL on chromosome 9: *qTL9‐1*. To further delimit the position of *qTL9‐1*, an F_2_ population containing 832 plants was created by self‐fertilizing an HIF that segregates only for *qTL9‐1* (Fig. S1b). The two markers that flanked *qTL9‐1*, M1 and M13, were first used to identify recombinants from the F_2_ population. A total of 110 recombinants were identified. Eleven additional markers were then developed to further resolve the breakpoints of the recombinants (Table S8). Based on the marker genotypes across the *qTL9‐1* region, the 110 recombinants can be classified into 10 genotype groups. Because of the limited field space, for each genotype group, only one recombinant family containing > 300 F_3_ seeds was selected and planted for progeny phenotyping. Following the previously described fine‐mapping procedure (Hung *et al*., 2012; Huang *et al*., 2016; Li *et al*., 2016), HR and HNR plants within each F_3_ family were identified and compared, to determine the genotype of gene in the parental recombinant (see the Materials and Methods section for details). Stepwise substitution mapping was then conducted across the 10 recombinant families to delimit the causal region of *qTL9‐1*.

**Figure 7:**
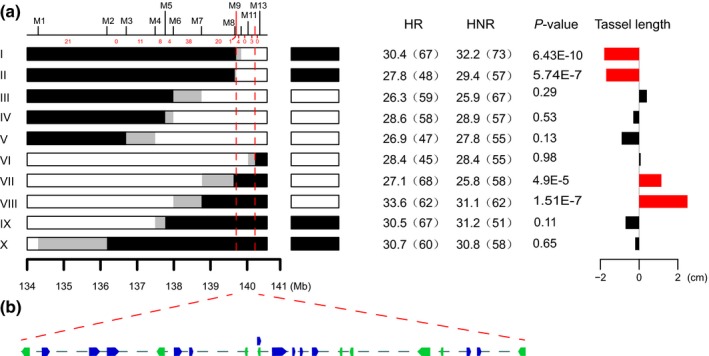
Fine mapping of *qTL9‐1*. (a) Delimiting the position of *qTL9‐1* with recombinants. The number of recombinants between markers is shown at the top. The recombinants identified in the F_2_ population were divided into 10 groups based on their genotypes. The graphical genotypes of the homozygous recombinants (HRs) within each recombinant group are shown on the left. Black or white boxes next to the HR indicate the corresponding homozygous nonrecombinants (HNRs) within each recombinant family. Black and white boxes indicate homozygous regions for the teosinte (*Zea mays* ssp. *parviglumis*) and maize (*Zea mays* ssp. *mays*) alleles, respectively. Gray boxes represent regions where recombination occurred. The values in the columns of HR and HNR are the phenotypic means, with sample size listed in parentheses. The graphs on the right indicate the phenotypic difference comparisons between the HR and HNR plants within each recombinant group. The allele effects are calculated as the tassel length difference between the HR and HNR plants. The red bars indicate a significant difference at *P *<* *0.01 after Bonferroni correction. The black bars indicate no significant difference between the HR and HNR plants. (b) Candidate genes in the fine‐mapped region. Blue and green rectangles represent 21 annotated genes in the reference genome, and the arrow indicates their transcription direction.

As shown in Fig. 7(a), genotype group X carried a recombination event between markers M1 and M2. No significant phenotypic difference in tassel length was detected between the HR and HNR plants, suggesting that the causal region of *qTL9‐1* is located downstream of marker M1. Group II carried a recombination event between markers M8 and M9. Significant phenotypic difference was detected between the HR and HNR plants, suggesting that the causal region of *qTL9‐1* is located downstream of marker M8. Using the same method, the location of the QTL effect was determined for each genotype group. The most informative comparisons are from groups I and VI which carried recombination events between M9 and M10 and between M11 and M12, respectively. Groups I and VI delimited *qTL9‐1* to a 513‐kb region between M9 and M12 that harbored 21 putative gene models (Fig. 7; Tables S2, S9).

## Discussion

Using a large maize‐teosinte population, the genetic architecture controlling the tassel morphological differences between maize and its wild progenitor, teosinte, was investigated. Our results showed that the five tassel traits examined in this study were associated with different genetic architecture features. TL and TBN appeared to be controlled by a single large‐effect QTL plus many small‐effect QTLs, whereas TBL, TPL and TBA were controlled by many small‐effect loci. The differences in genetic architecture characteristics between tassel traits might reflect their different evolutionary histories. Traits that have a long evolutionary history and are important for both maize and teosinte should have genetic architectures with a very large number of genes each contributing a small amount to the phenotype (Wallace *et al*., 2014). Any large‐effect alleles for such traits would presumably have been fixed rapidly (Orr, 1998). Traits that came under strong and recent selection during and after domestication should have architectures with fewer genes and/or larger effect sizes because of the shorter amount of time for variation to accumulate (Wallace *et al*., 2014). TL and TBN, which are major factors determining tassel size, are major selection traits for the consistently decreasing tassel size during maize domestication and improvement. Therefore, their evolution was probably initiated by the selection of a few major mutations and the further refinement of many modifier loci with small effects. The traits TBL, TPL and TBA are factors defining the spatial morphology of tassels. No consistent trait change trend was found in them during maize domestication and improvement (Duvick, 2005), and they thus probably evolved through a series of incremental mutations of small effects.

To evaluate whether the QTLs identified in our maize‐teosinte population were also detected in maize populations, we compared the QTLs detected in this maize‐teosinte BC_2_S_3_ population with the largest tassel QTL mapping study, which was recently performed by Wu *et al*. (2016), who used nearly 8000 maize RILs and inbred lines. Of the 25 TL and TBN QTLs mapped in this study, 14 (56%) QTLs overlapped with the QTLs reported by Wu *et al*. (2016) and 11 (44%) QTLs were specific to our maize‐teosinte population (Table S4). The commonly mapped QTLs suggested that there are common genetic variants contributing to tassel variation in maize‐teosinte and maize‐maize cross populations. The QTL mapping inconsistencies might be caused by differences in the genetic design and statistical approaches employed in the two studies. It is interesting to note that the specifically mapped QTLs in our maize‐teosinte population might be loci important for tassel domestication. For example, the major‐effect QTL for TBN, *qTBN7‐1*, overlapped with a TBN QTL mapped in the joint‐linkage analysis of the CN NAM populations (11 RIL populations) but not with a TBN QTL in the joint‐linkage analysis of the US NAM populations (25 RIL populations). Because of its close position to the QTL peak, strong selection signal and developmental role, we speculate that *ra1* is probably the underlying gene for *qTBN7‐1*. However, significant associations were not detected in the *ra1* region in the genome‐wide association mapping of TBN in the study of Wu *et al*. (2016), who used a panel of 945 maize inbred lines that represented the global maize genetic diversity. These results suggest that the causal *ra1* variant might be nearly fixed or remain at a low allele frequency in maize populations, thereby resulting in the low detection power in maize populations. Therefore, further identification of the causal variant of *ra1* and in‐depth population genetic analyses in maize and teosinte populations are required to obtain more direct insights into the role of *ra1* in controlling maize tassel domestication.

We demonstrated that the known maize inflorescence development genes were significantly enriched in tassel QTL regions, and many of these genes showed significant signals of selection, suggesting that they might play important roles in driving maize tassel evolution. In addition to the inflorescence development genes, we also showed that other types of genes, such as flowering time genes, might be involved in the regulation of tassel natural variation. An association analysis demonstrated that the causal CACTA‐like TE at the *ZmCCT* promoter for photoperiod sensitivity was also strongly associated with tassel size traits. The early flowering lines that carry the TE insertion were associated with reduced TBN and TL. One simple explanation for this might be that plants with earlier flowering time have less time to grow and photosynthesize and hence have less energy available to fuel the development of reproductive organs. However, increasing evidence has indicated that flowering time genes might act as important timing signals to regulate inflorescence development. *Grain number*,* plant height*, and *heading date7* (*Ghd7*), the *ZmCCT* homolog in rice (*Oryza sativa*), also showed a pleiotropic effect on panicle branching and functioned as an integrator that linked floral transition and lateral branch development (Xue *et al*., 2008; Weng *et al*., 2014). Two key flowering time genes in rice, *Heading date 1* (*Hd1*) and *Early heading date 1* (*Ehd1*), were found to cooperatively control panicle development by regulating the expression of the florigen genes *Hd3a* and *Rice Flowering‐locus T 1* (*RFT1*) (Endo‐Higashi & Izawa, 2011). An inverse relationship between the number of inflorescence branches and the strength of the floral promoting signal was observed in rice (Endo‐Higashi & Izawa, 2011). In Arabidopsis, the two florigen genes *FLOWERING LOCUS T* (*FT*) and *TWIN SISTER OF FT* (*TSF*) were also shown to modulate lateral shoot development (Hiraoka *et al*., 2013). Similarly, *Photoperiod‐1* (*Ppd‐1*), a pseudo‐response regulator gene that controls photoperiod‐dependent floral induction in wheat (*Triticum aestivum*) (Turner *et al*., 2005), has recently been shown to play a crucial role in regulating inflorescence architecture and paired spikelet development by modulating the strength of the floral promoting signal (FT) during early reproductive development (Boden *et al*., 2015). A weak floral signal slightly delays the conversion of inflorescence axillary meristems to spikelet meristems, thus facilitating the formation of paired spikelets. It is interesting to note that the FT‐like floral activator gene *ZCN8* also co‐localized with a QTL for TL identified in this study, although the causal link requires further study. These results strongly suggest that flowering time genes may play an important role in modulating inflorescence variation by controlling the timing of the conversion between meristems during inflorescence development.

Using the NILs developed from the HIF, we narrowed down a major‐effect QTL for tassel length, *qTL9‐1*, to a 513‐kb physical region. Based on the gene annotation of the B73 reference genome (Schnable *et al*., 2009), there are 21 genes located in the 513‐kb target region (Tables S2, S9). Among these genes, *GRMZM2G098784* encodes a SCARECROW (SCR) subfamily GRAS (GAI, RGA and SCARECROW) transcription factor. GRAS transcription factors are important regulators involved in the control of various biological processes, such as hormone signaling and meristem maintenance and development (Bolle, 2004). *SCARECROW* (*AtSCR*) was the first member of the GRAS family identified in Arabidopsis. The loss of function of *AtSCR* leads to aberrant root growth and impaired inflorescence development (Di Laurenzio *et al*., 1996). *GRMZM2G053199* encodes a ser/thr protein phosphatase family protein, and its Arabidopsis ortholog, *Type‐One Protein Phosphatase4* (*TOPP4*), controls the degradation of the DELLA protein in the gibberellin (GA) signaling pathway (Qin *et al*., 2014). The mutation of *TOPP4* results in a dwarfed phenotype, whereas the overexpression of *TOPP4* leads to increased plant height, thickened stems, and enlarged rosette leaves and inflorescences (Qin *et al*., 2014). *GRMZM2G492252* encodes a RING finger E3 ligase, and its Arabidopsis ortholog is involved in abscisic acid‐related stress signal transduction (Zhang *et al*., 2007). Further fine‐mapping and functional experiments are required to identify the underlying gene for *qTL9‐1*.

## Author contributions

F.T. and G.X. designed the research. G.X., X.W., C.H., D.X., D.L., J.T., Q.C., C.W., Y.L. and Y.W. conducted fieldwork. G.X. and X.W. performed molecular experiments and analyzed data. X.Y. performed candidate gene association analysis. F.T. and G.X. wrote the manuscript. All authors read and approved the final manuscript.

## Supporting information

Please note: Wiley Blackwell are not responsible for the content or functionality of any Supporting Information supplied by the authors. Any queries (other than missing material) should be directed to the *New Phytologist* Central Office.

**Fig. S1** Graphical genotype of a heterogeneous inbred family used to construct the near‐isogenic lines.
**Fig. S2** Inflorescence gene enrichment analysis in tassel QTL regions.
**Table S1** List of known inflorescence development‐related genes in maize
**Table S2** Gene identifiers used in this study
**Table S3** Maize and teosinte materials used for *bif2* sequencing
**Table S4** QTLs for TL, TBL, TPL, TBN, and TBA identified in the maize‐teosinte BC_2_S_3_ RIL population
**Table S5** Phenotypic effect analyses of NILs at the *ra1* locus on chromosome 7
**Table S6** Primers used for *bif2* sequencing
**Table S7** Nucleotide diversity statistics at *bif2*
**Table S8** Molecular markers used for QTL effect validation and *qTL9‐1* fine mapping
**Table S9** Functional annotation of the 21 candidate genes in the 513‐kb target region of *qTL9‐1*
