# Supplementary material for: Complex genetic architecture underlies maize tassel domestication
Source: New Phytol. 2017 Jan 9;214(2):852–64. doi: 10.1111/nph.14400 (PMC5363343; doi:10.1111/nph.14400)
Supplement: Supplementary file 1 — Fig. S1 Graphical genotype of a heterogeneous inbred family used to construct the near‐isogenic lines. Fig. S2 Inflorescence gene enrichment analysis in tassel QTL regions. Table S1 List of known inflorescence development‐related genes in maize Table S2 Gene identifiers used in this study Table S3 Maize and teosinte materials used for bif2 sequencing Table S4 QTLs for TL, TBL, TPL, TBN, and TBA identified in the maize‐teosinte BC2S3 RIL population Table S5 Phenotypic effect analyses of NILs at the ra1 locus on chromosome 7 Table S6 Primers used for bif2 sequencing Table S7 Nucleotide diversity statistics at bif2 Table S8 Molecular markers used for QTL effect validation and qTL9‐1 fine mapping Table S9 Functional annotation of the 21 candidate genes in the 513‐kb target region of qTL9‐1 [file NPH-214-852-s001.pdf]

New Phytologist

New Phytologist **Supporting Information Figs S1 & S2 and Tables S1–S9**

Article title: Complex genetic architecture underlies maize tassel domestication

Authors: Guanghui Xu, Xufeng Wang, Cheng Huang, Dingyi Xu, Dan Li, Jinge Tian, Qiuyue Chen, Chenglong Wang, Yameng Liang, Yaoyao Wu, Xiaohong Yang and Feng Tian

Article acceptance date: 16 November 2016

The following items of Supporting Information are available for this article:

Fig. S1 Graphical genotype of a heterogeneous inbred family used to construct the near-isogenic lines.

Fig. S2 Inflorescence gene enrichment analysis in tassel QTL regions.

Table S1 List of known inflorescence development related genes in maize

Table S2 Gene identifiers used in this study

Table S3 Maize and teosinte materials used for *bif2* sequencing

Table S4 QTLs for TL, TBL, TPL, TBN, and TBA identified in the maize-teosinte BC_2S_3 RIL population

Table S5 Phenotypic effect analyses of NILs at *ra1* locus on chromosome 7

Table S6 Primers used for *bif2* sequencing

Table S7 Nucleotide diversity statistics at *bif2*

Table S8 Molecular markers used for QTL effect validation and *qTL9-1* fine mapping

Table S9 Functional annotation of the 21 candidate genes in the 513-kb target region of *qTL9-1*

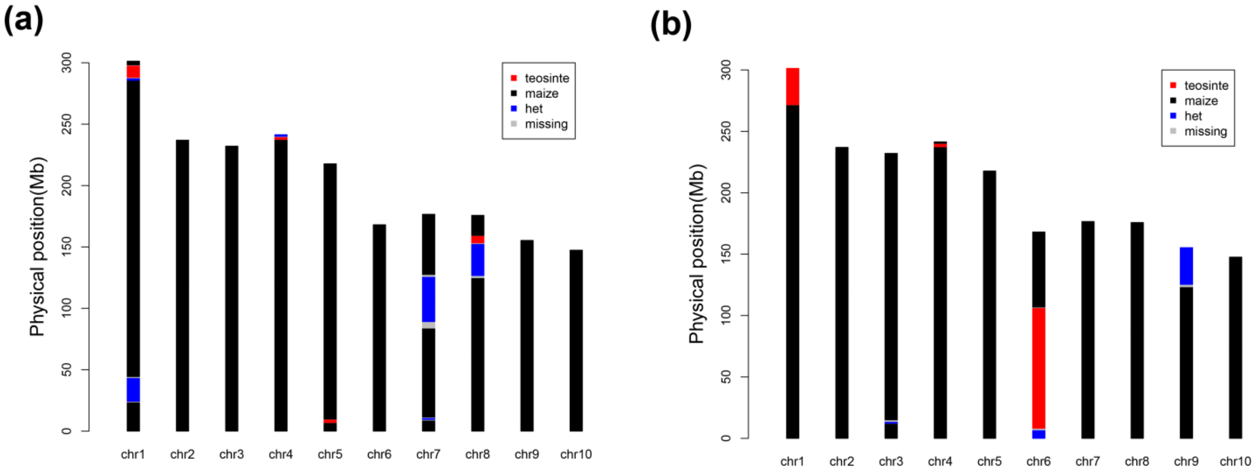

Fig. S1 Graphical genotype of a heterogeneous inbred family (HIF) used to construct the near-isogenic lines. (a) HIF used to construct NILs for validating the effect of *ra1*. (b) HIF used to construct NILs for *qTL9-1*. Black, regions homozygous for the W22 allele; red, regions homozygous for the teosinte allele; blue, heterozygous regions; grey, unknown regions.

New Phytologist

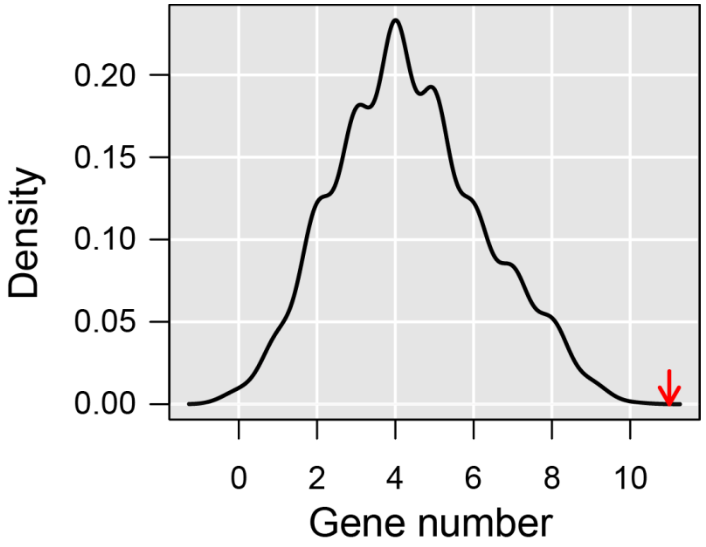

Fig. S2 Inflorescence gene enrichment analysis in tassel QTL regions. Details of the permutation test are described in the Materials and Methods section. The density plot shows the distribution of the number of inflorescence genes that resided in the simulated QTL region in each permutation. The red arrow indicates the observed number of inflorescence genes that co-localized with tassel QTLs.

New Phytologist

Table S1 List of known inflorescence development related genes in maize

Gene	Gene model	Annotation	Chr	Gene start (bp)*	Gene end (bp)*	Mutant phenotypes in the tassel	Reference
dcl1	GRMZM2G040762	dicer-like 1	1	4,607,185	4,614,592	Unrecognizable glumes;extra florets; abnormal stamens	Thompson *et al.* (2014)
ts2	GRMZM2G455809	oxidoreductase	1	46,678,942	46,680,363	Conversion from staminate to pistillate	DeLong *et al.* (1993)
rte	GRMZM2G166159	boron transporter protein	1	149,312,438	149,317,160	Shorter and less branched tassel; devoid of spikelets	Chatterjee *et al.* (2014)
bif2	GRMZM2G171822	protein kinase	1	173,846,756	173,848,499	Decreased tassel branches and spikelets	McSteen & Hake (2001)
ub2	GRMZM2G160917	SBP-box family protein	1	188,181,863	188,185,970	Decreased tassel branch number	Chuck *et al.* (2014)
tls1	GRMZM2G176209	aquaporin protein	1	223,842,177	223,846,120	Small tassels or lack a tassel	Leonard *et al.* (2014)
kn1	GRMZM2G017087	homeobox domain containing protein	1	271,340,805	271,348,525	Less tassel branches and spikelets	Bolduc *et al.* (2012)
ids1	GRMZM5G862109	AP2 domain containing protein	1	292,889,740	292,893,983	Additional florets in tassel; larger spikelets in tassel	Chuck *et al.* (1998)
zfl2	GRMZM2G180190	transcription factor FL	2	12,641,797	12,644,804	Decreased tasse long branches	Bomblies *et al.* (2003)
ts1	GRMZM2G104843	lipoxygenase protein	2	45,192,092	45,196,460	Conversion from staminate to pistillate in the tassel	Acosta *et al.* (2009)
BAD1	GRMZM2G110242	TCP family TF	2	179,980,551	179,982,009	Acute tassel branch angles	Bai *et al.* (2012)
ra2	AC233943.1_FG002	LOB-domain TF	3	12,882,491	12,883,276	Branches becoming increasingly shorter toward the inflorescence apex; longer pedicel; more accute tassel branch	Bortiri *et al.* (2006)
fea3	GRMZM2G166524	LRR receptor	3	29,094,519	29,097,953	Thick tassels; enlarged meristems	Je *et al.* (2016)
ts4	GRMZM5G803935	mir172	3	144,884,032	144,884,741	Male floral organs failed to develop; increased meristem branching	Chuck *et al.* (2007)
na1	GRMZM2G449033	steroid reductase	3	178,991,745	178,993,461	Feminized male flower	Hartwig *et al.* (2011)
ba1	GRMZM2G397518	HLH domain containing protein	3	183,084,299	183,085,268	Shortened and unbranched tassel; sterile floret	Gallavotti *et al.* (2004)
fea2	GRMZM2G104925	LRR family	4	133,662,510	133,664,998	Wider rachis; increased spikelet density	Taguchi-Shiobara *et al.* (2001)
tu1	GRMZM2G370777	MADS-box family protein	4	178,887,359	178,894,404	Elongated outer glumes in male floral organ	Han *et al.* (2012)
ub3	GRMZM2G460544	SBP-box family protein	4	199,278,823	199,283,117	Decreased tassel branch number	Chuck *et al.* (2014)
td1	GRMZM2G300133	leucine-rich receptor-like protein kinase	5	61,670,412	61,673,965	Increased spilelet density; more stamens	Bommert *et al.* (2005b)
fea4	GRMZM2G133331	bZIP TF	6	116,751,859	116,754,624	Fasciated tassel; thicker main rachis in tassel; increased spikelet density	Pautler *et al.* (2015)
BIF4	GRMZM5G864847	Aux/IAA family protein	6	130,004,758	130,006,167	Reduced tassel branches and spikelets	Galli *et al.* (2015)
tsh1	GRMZM2G325850	zinc finger TF	6	166,077,055	166,078,403	Reduced branching; brack growth	Whipple *et al.* (2010)
sid1	GRMZM2G176175	AP2 family protein	7	22,004,372	22,015,553	Fewer tassel branches (double mutant with ids1)	Chuck *et al.* (2008)
ra1	GRMZM2G003927	zinc finger domain containing protein	7	110,331,505	110,332,253	More tassel branches	Vollbrecht *et al.* (2005)
tsh4	GRMZM2G307588	SBP-box family protein	7	133,170,018	133,173,665	Less branched; extra leaves at the base of the tassel	Chuck *et al.* (2010)
ra3	GRMZM2G014729	glycosyl hydrolase	7	166,856,424	166,860,909	More tassel branches	Satoh-Nagasawa *et al.* (2006)
bd1	GRMZM2G307119	AP2 domain containing protein	7	172,207,717	172,209,213	Fascinated lateral spikelets in the tassel;	Chuck *et al.* (2002)
BIF1	GRMZM2G130953	Aux/IAA family protein	8	18,318,828	18,320,919	Reduced tassel branches and spikelets	Galli *et al.* (2015)
baf1	GRMZM2G072274	AT-hook protein	9	21,965,527	21,967,096	More compact tassel; increased tassel branch number, shorter tassel branch	Gallavotti *et al.* (2011)
zfl1	GRMZM2G098813	transcription factor FL	10	140,851,443	140,854,305	Increased tassel long branches	Bomblies *et al.* (2003)

* The physical positions of genes are according to maize B73 reference genome AGPv2

New Phytologist

Table S2 Gene identifiers used in this study

V2 Gene ID	V4 Gene ID	Chr	V2 Start	V2 End	V4 Start	V4 End
GRMZM2G040762	Zm00001d027412	1	4,607,185	4,614,592	4,724,456	4,736,832
GRMZM2G455809	Zm00001d028806	1	46,678,942	46,680,363	46,955,327	46,956,671
GRMZM2G166159	Zm00001d030656	1	149,312,438	149,317,160	151,221,229	151,225,821
GRMZM2G171822		1	173,846,756	173,848,499		
GRMZM2G160917	Zm00001d031451	1	188,181,863	188,185,970	190,383,261	190,386,589
GRMZM2g176209	Zm00001d032461	1	223,842,177	223,846,120	227,449,316	227,453,079
GRMZM2G017087	Zm00001d033859	1	271,340,805	271,348,525	276,073,335	276,081,242
GRMZM5G862109	Zm00001d034629	1	292,889,740	292,893,983	298,422,859	298,427,050
GRMZM2G180190	Zm00001d002449	2	12,641,797	12,644,804	12,914,091	12,917,068
GRMZM2G104843	Zm00001d003533	2	45,192,092	45,196,460	47,105,187	47,109,372
GRMZM2G110242	Zm00001d005737	2	179,980,551	179,982,009	185,422,359	185,423,189
AC233943.1_FG002	Zm00001d039694	3	12,882,491	12,883,276	12,158,280	12,159,065
GRMZM2G166524	Zm00001d040130	3	29,094,519	29,097,953	28,711,131	28,713,722
GRMZM5G803935		3	144,884,032	144,884,741		
GRMZM2G449033	Zm00001d042843	3	178,991,745	178,993,461	181,821,465	181,822,989
GRMZM2G397518	Zm00001d042989	3	183,084,299	183,085,268	186,014,629	186,015,264
GRMZM2G104925	Zm00001d051012	4	133,662,510	133,664,998	136,765,871	136,767,712
GRMZM2G370777	Zm00001d052180	4	178,887,359	178,894,404	181,858,166	181,863,932
GRMZM2G460544	Zm00001d052890	4	199,278,823	199,283,117	203,611,347	203,615,518
GRMZM2G300133	Zm00001d014793	5	61,670,412	61,673,965	63,456,839	63,460,120
GRMZM2G133331	Zm00001d037317	6	116,751,859	116,754,624	120,724,112	120,726,773
GRMZM5G864847	Zm00001d037691	6	130,004,758	130,006,167	134,088,831	134,092,670
GRMZM2G325850	Zm00001d039113	6	166,077,055	166,078,403	170,248,013	170,249,485
GRMZM2G176175	Zm00001d019230	7	22,004,372	22,015,553	23,054,461	23,065,589
GRMZM2G003927	Zm00001d020430	7	110,331,505	110,332,253	113,572,410	113,572,937
GRMZM2G307588	Zm00001d020941	7	133,170,018	133,173,665	137,273,600	137,277,139
GRMZM2G014729	Zm00001d022193	7	166,856,424	166,860,909	172,484,959	172,489,194
GRMZM2G307119	Zm00001d022488	7	172,207,717	172,209,213	178,605,958	178,606,905
GRMZM2G130953	Zm00001d008749	8	18,318,828	18,320,919	18,951,758	18,953,833
GRMZM2G072274	Zm00001d045427	9	21,965,527	21,967,096	21,786,350	21,787,375
GRMZM2G103270	Zm00001d047806	9	139,698,112	139,701,345	142,285,993	142,289,465
GRMZM2G103247	Zm00001d047807	9	139,705,351	139,707,757	142,293,225	142,295,467
GRMZM2G063151	Zm00001d047808	9	139,744,606	139,748,920	142,331,350	142,336,894
GRMZM2G114126	Zm00001d047812	9	139,761,157	139,765,745	142,388,915	142,393,403
GRMZM2G098784	Zm00001d047813	9	139,836,105	139,839,851	142,473,442	142,477,283
GRMZM2G151992	Zm00001d047814	9	139,844,221	139,846,531	142,497,782	142,500,049
GRMZM2G700034		9	139,854,927	139,855,509		
GRMZM2G103595	Zm00001d047820	9	139,929,569	139,930,729	142,587,964	142,588,263
GRMZM2G064710	Zm00001d047821	9	139,942,775	139,945,257	142,594,236	142,596,825
AC202144.5_FG001	Zm00001d047822	9	139,943,334	139,945,062	142,595,493	142,596,552
GRMZM2G492252		9	139,947,777	139,955,628		
GRMZM2G045084	Zm00001d047824	9	139,961,781	139,963,488	142,612,911	142,613,747
GRMZM2G045102	Zm00001d047827	9	139,968,673	139,969,693	142,619,272	142,620,292
GRMZM5G888263	Zm00001d047828	9	139,980,251	139,983,891	142,625,874	142,631,952

V2 Gene ID	V4 Gene ID	Chr	V2 Start	V2 End	V4 Start	V4 End
GRMZM2G145313	Zm00001d047829	9	140,014,798	140,016,394	142,660,881	142,662,320
GRMZM2G089803	Zm00001d047830	9	140,023,280	140,026,450	142,669,294	142,672,793
GRMZM2G164160	Zm00001d047833	9	140,111,984	140,120,409	142,784,493	142,792,921
GRMZM2G164136	Zm00001d047834	9	140,123,391	140,127,499	142,795,903	142,800,053
GRMZM2G053199	Zm00001d047835	9	140,162,005	140,163,880	142,826,331	142,832,989
GRMZM2G700036		9	140,166,647	140,167,741		
GRMZM2G151934	Zm00001d047837	9	140,212,757	140,217,688	142,882,570	142,887,070
GRMZM2G098813	Zm00001d026231	10	140,851,443	140,854,467	141,561,862	141,564,767

Table S3 Maize and teosinte materials used for *bif2* sequencing

Material	Taxon	Location of the teosinte accessions	Source	A1	A2	A3	A4	A5	A6	A7
TIL11 (Ames 28405)	*Zea mays* ssp. *Parviglumis*	Huitzuco, Guerrero	John Doebley[a]			√	√			√
TIL17 (Ames 28409)	*Zea mays* ssp. *Parviglumis*	Teloloapan	John Doebley[a]		√	√	√	√	√	√
TIL01 (Ames 28399)	*Zea mays* ssp. *Parviglumis*	Tzitzio, Michoacan	John Doebley[a]	√	√	√		√	√	
TIL15 (Ames 28407)	*Zea mays* ssp. *Parviglumis*	Palo Blanco	John Doebley[a]			√			√	√
TIL16 (Ames 28408)	*Zea mays* ssp. *Parviglumis*	Palo Blanco	John Doebley[a]		√	√			√	
TIL10 (Ames 28404)	*Zea mays* ssp. *Parviglumis*	Teloloapan	John Doebley[a]			√			√	√
TIL06 (Ames 28401)	*Zea mays* ssp. *Parviglumis*	Palo Blanco	John Doebley[a]	√		√				
TIL03 (Ames 28400)	*Zea mays* ssp. *Parviglumis*	La Lima, Jalisco	John Doebley[a]	√		√	√	√	√	
TIL14 (Ames 28406)	*Zea mays* ssp. *Parviglumis*	Rodeo	John Doebley[a]			√			√	
TIL09 (Ames 28403)	*Zea mays* ssp. *Parviglumis*	Tejupilco, Mexico	John Doebley[a]	√	√			√	√	
PI 566692	*Zea mays* ssp. *Parviglumis*	Tuzantla	NPGS		√	√	√	√		√
PI 384063	*Zea mays* ssp. *Parviglumis*	Valle de Bravo, Mexico	NPGS			√			√	√
PI 384069	*Zea mays* ssp. *Parviglumis*	El Pochote	NPGS	√				√		√
PI 566686	*Zea mays* ssp. *Parviglumis*	Paso de Morelos, Mexico	NPGS				√	√		√
PI 384062	*Zea mays* ssp. *Parviglumis*	Palo Blanco	NPGS	√	√	√	√			√
Ames 21797	*Zea mays* ssp. *Parviglumis*	Mazatlan/El Salado	NPGS	√	√	√	√			√
Ames 21889	*Zea mays* ssp. *Parviglumis*	El Rodeo	NPGS			√	√			√
Ames 21861	*Zea mays* ssp. *Parviglumis*	Unknown	NPGS			√			√	√
Ames 21826	*Zea mays* ssp. *Parviglumis*	El Salado	NPGS						√	√
CIMMYT ID13582	*Zea mays* ssp. *Parviglumis*	Guerrero, Mexico	Xiaohong Yang[b]	√	√	√	√	√	√	√
CIMMYT 8759	*Zea mays* ssp. *Parviglumis*	Unknown	CIMMYT		√	√	√		√	
B73	*Zea mays* ssp. *mays*			√	√	√	√	√	√	√
Mo17	*Zea mays* ssp. *mays*			√	√	√	√	√	√	
W22	*Zea mays* ssp. *mays*				√	√	√	√	√	
B97	*Zea mays* ssp. *mays*			√	√	√		√	√	√
CML103	*Zea mays* ssp. *mays*			√	√	√	√	√	√	√
CML228	*Zea mays* ssp. *mays*			√	√	√		√	√	√
CML247	*Zea mays* ssp. *mays*			√	√	√		√	√	√
CML277	*Zea mays* ssp. *mays*			√	√	√		√	√	√
CML322	*Zea mays* ssp. *mays*					√	√	√	√	√
CML333	*Zea mays* ssp. *mays*			√	√	√		√	√	√
CML52	*Zea mays* ssp. *mays*			√	√	√		√	√	√
CML69	*Zea mays* ssp. *mays*			√	√	√	√	√	√	√
Hp301	*Zea mays* ssp. *mays*				√	√				
Il14H	*Zea mays* ssp. *mays*			√	√	√		√		√
Ki11	*Zea mays* ssp. *mays*			√	√	√	√	√	√	√
Ki3	*Zea mays* ssp. *mays*			√	√	√		√	√	√
Ky21	*Zea mays* ssp. *mays*			√	√	√		√	√	
M162W	*Zea mays* ssp. *mays*			√	√	√	√	√	√	√
M37W	*Zea mays* ssp. *mays*			√	√	√	√	√	√	√
Mo18W	*Zea mays* ssp. *mays*			√	√	√		√	√	√
MS71	*Zea mays* ssp. *mays*			√	√	√		√	√	
NC350	*Zea mays* ssp. *mays*			√	√	√		√	√	

Material	Taxon	Location of the teosinte accessions	Source	A1	A2	A3	A4	A5	A6	A7
NC358	*Zea mays* ssp. *mays*			√	√	√		√	√	
Oh43	*Zea mays* ssp. *mays*			√	√	√	√	√	√	
Oh7B	*Zea mays* ssp. *mays*			√	√	√	√	√	√	√
P39	*Zea mays* ssp. *mays*			√	√	√		√	√	√
Tx303	*Zea mays* ssp. *mays*			√	√	√		√		√
Tzi8	*Zea mays* ssp. *mays*			√	√	√	√	√	√	√

[a]Gift from John Doebley (University of Wisconsin, Madison WI). [b]Gift from Xiaohong Yang (China Agricultural University, Bejing, China).

NPGS, the USDA National Plant Germplasm System

New Phytologist

Table S4 QTLs for TL, TBL, TPL, TBN, and TBA identified in the maize-teosinte BC_2S_3 RIL population

Traits	Chr	QTL	LOD	Var (%)	Additive effect[a]	Dominance effect	Peak position (cM)	Genetic interval (cM)[b]	Peak position (bp)	Physical Interval (bp)[c]	Overlapped QTLs in Wu et al. (2016)[d]
TBA	1	qTBA1-1	10.3	2.9	-1.57	0.49	30.7	27.0 - 35.0	12,852,915	12,165,978 - 16,067,694	NA
TBA	1	qTBA1-2	13.0	3.7	-1.62	-0.74	89.5	88.0 - 90.5	173,846,135	164,493,706 - 174,526,566	NA
TBA	1	qTBA1-3	4.6	1.3	-0.92	-0.29	167.7	156.8 - 174.9	291,863,107	285,214,192 - 295,261,584	NA
TBA	2	qTBA2-1	13.6	3.9	-1.44	-1.72	9.0	4.5 - 12.3	2,619,855	1,972,797 - 3,565,356	NA
TBA	2	qTBA2-2	6.7	1.9	-1.57	0.90	45.4	41.5 - 49.7	13,862,153	12,129,937 - 20,401,964	NA
TBA	2	qTBA2-3	5.8	1.6	-1.28	0.72	89.2	87.7 - 95.0	178,134,722	175,369,168 - 184,401,600	NA
TBA	3	qTBA3-1	8.3	2.3	1.70	-0.83	75.5	73.5 - 76.4	156,963,575	150,049,465 - 158,451,370	NA
TBA	4	qTBA4-1	5.6	1.6	-1.24	0.42	77.1	74.0 - 84.5	157,767,208	152,413,237 - 166,870,401	NA
TBA	5	qTBA5-1	6.5	1.8	1.19	-0.17	89.5	88.6 - 92.0	163,890,034	160,656,627 - 167,663,779	NA
TBA	6	qTBA6-1	5.0	1.4	-0.96	-0.19	8.5	6.2 - 15.0	9,206,228	6,319,324 - 33,622,200	NA
TBA	6	qTBA6-2	12.8	3.7	-1.74	0.17	78.6	77.7 - 80.4	153,228,215	152,193,604 - 154,331,012	NA
TBA	7	qTBA7-1	7.6	2.2	1.07	0.84	24.0	18.1 - 34.0	8,049,850	5,960,740 - 12,987,006	NA
TBA	7	qTBA7-2	8.6	2.4	1.36	0.05	112.4	108.0 - 114.5	161,910,993	160,525,223 - 162,566,314	NA
TBA	7	qTBA7-3	5.8	1.6	1.07	-0.56	139.5	133.6 - 150.1	173,041,532	171,789,940 - 176,600,018	NA
TBA	8	qTBA8-1	6.5	1.8	1.22	-0.31	61.4	52.2 - 63.8	67,243,908	21,615,617 - 86,403,088	NA
TBA	9	qTBA9-1	5.5	1.5	-1.17	0.92	15.6	5.9 - 21.0	10,468,151	4,507,564 - 11,774,963	NA
TBA	10	qTBA10-1	14.0	4.0	-2.50	-0.20	43.7	41.1 - 45.1	117,568,031	109,717,908 - 124,069,452	NA
TBA	10	qTBA10-2	7.0	2.0	-1.35	1.01	57.8	55.6 - 80.5	142,785,958	142,358,481 - 144,529,385	NA
TBL	1	qTBL1-1	7.6	1.9	0.54	-0.43	2.1	1.0 - 6.5	1,780,255	6,977 - 3,647,376	NA
TBL	1	qTBL1-2	13.3	3.3	0.57	0.14	59.7	53.4 - 60.5	34,528,556	30,685,696 - 34,946,656	NA
TBL	1	qTBL1-3	10.0	2.5	0.70	-0.19	92.4	88.6 - 95.3	180,406,301	165,204,778 - 203,717,542	NA
TBL	1	qTBL1-4	4.4	1.1	0.23	0.66	113.3	110.5 - 117.5	227,451,739	224,101,561 - 230,882,238	NA
TBL	1	qTBL1-5	11.1	2.7	1.08	-0.52	125.2	124.9 - 126.3	249,454,565	248,858,801 - 251,157,209	NA
TBL	2	qTBL2-1	6.7	1.6	-0.54	0.44	66.8	65.2 - 69.3	38,099,465	36,452,974 - 39,426,884	NA
TBL	3	qTBL3-1	11.7	2.9	-0.66	-0.06	37.0	33.9 - 37.7	11,006,846	9,428,671 - 11,213,530	NA
TBL	3	qTBL3-2	7.0	1.7	0.62	0.01	65.7	64.1 - 69.5	90,026,994	60,571,016 - 138,132,306	NA
TBL	4	qTBL4-1	10.6	2.6	0.57	0.08	101.9	98.2 - 105.0	180,375,774	178,062,732 - 181,871,720	NA
TBL	5	qTBL5-1	4.7	1.1	0.40	-0.09	1.0	1.0 - 3.7	187,261	187,261 - 1,960,072	NA
TBL	5	qTBL5-2	9.3	2.3	0.38	0.46	165.9	164.1 - 169.5	214,499,583	214,163,665 - 215,358,824	NA
TBL	7	qTBL7-1	21.3	5.4	-0.66	-0.37	59.2	56.3 - 61.9	112,974,666	105,763,843 - 120,108,945	NA
TBL	7	qTBL7-2	6.7	1.6	-0.44	0.10	124.2	121.5 - 127.9	168,290,368	167,622,045 - 169,748,915	NA
TBL	7	qTBL7-3	4.3	1.0	-0.34	0.22	148.5	142.5 - 150.1	175,567,526	173,486,731 - 176,600,018	NA
TBL	8	qTBL8-1	15.3	3.8	-0.64	-0.03	58.6	56.4 - 61.5	26,751,960	24,720,800 - 68,938,289	NA
TBL	9	qTBL9-1	24.2	6.2	0.90	0.04	78.5	76.5 - 79.6	131,382,793	129,185,361 - 133,546,599	NA
TBL	10	qTBL10-1	19.2	4.8	1.24	-0.32	39.5	38.5 - 40.8	99,473,184	88,407,521 - 106,051,092	NA
TBN	1	qTBN1-1	9.7	2.8	1.34	0.69	44.3	39.3 - 47.2	22,383,982	18,584,005 - 24,931,711	Q26CN-NAM
TBN	1	qTBN1-2	12.0	3.5	1.41	1.50	87.6	83.7 - 95.3	163,154,470	60,087,839 - 202,169,246	Q40US-NAM Q41US-NAM Q27CN-NAM Q28CN-NAM
TBN	2	qTBN2-1	9.7	2.8	1.83	0.20	17.0	15.1 - 18.5	4,850,467	4,178,651 - 5,076,659	Q45US-NAM Q31CN-NAM

New Phytologist

Traits	Chr	QTL	LOD	Var (%)	Additive effect[a]	Dominance effect	Peak position (cM)	Genetic interval (cM)[b]	Peak position (bp)	Physical Interval (bp)[c]	Overlapped QTLs in Wu et al. (2016)[d]
TBN	2	qTBN2-2	10.4	3.0	2.57	-0.33	125.9	123.9 - 126.7	215,089,211	213,870,961 - 217,406,822	Q34CN-NAM
TBN	2	qTBN2-3	5.3	1.5	0.94	0.80	174.5	162.1 - 177.2	236,471,027	233,911,314 - 236,971,678	Q50US-NAM
TBN	3	qTBN3-1	8.6	2.5	1.42	0.72	93.5	91.1 - 98.5	176,434,787	173,380,753 - 179,262,887	Q52US-NAM
TBN	5	qTBN5-1	5.9	1.7	0.69	1.96	108.6	106.0 - 120.8	186,678,599	183,518,368 - 194,092,278	Q59US-NAM<br>Q41CN-NAM
TBN	6	qTBN6-1	11.2	3.2	1.37	1.20	13.5	9.9 - 14.9	27,853,443	22,911,163 - 32,461,416	
TBN	6	qTBN6-2	19.6	5.8	2.18	0.63	76.3	73.4 - 77.9	150,889,358	147,346,417 - 152,316,833	Q44CN-NAM
TBN	6	qTBN6-3	5.3	1.5	1.15	-0.44	110.4	99.1 - 110.4	168,067,428	163,180,162 - 168,067,428	
TBN	7	qTBN7-1	42.4	13.4	3.30	-0.79	58.0	57.1 - 59.2	109,726,832	108,481,524 - 112,974,666	Q45CN-NAM
TBN	8	qTBN8-1	4.3	1.2	-1.04	0.54	88.6	79.9 - 94.4	148,898,581	129,979,319 - 154,678,274	Q67US-NAM
TBN	9	qTBN9-1	6.0	1.7	-1.25	1.24	35.4	31.6 - 37.3	20,228,529	18,335,150 - 20,607,456	
TBN	10	qTBN10-1	8.4	2.4	2.21	0.30	39.4	38.5 - 41.8	91,669,656	87,097,012 - 112,587,292	Q52CN-NAM
TL	1	qTL1-1	14.6	4.6	0.42	1.76	113.3	111.7 - 113.9	226,920,999	225,123,043 - 228,056,826	
TL	1	qTL1-2	6.2	1.9	1.18	-1.11	125.6	123.8 - 129.0	249,530,926	246,201,212 - 255,638,073	Q5US-NAM
TL	3	qTL3-1	9.2	2.9	-0.81	0.04	25.7	23.1 - 30.5	6,523,504	5,886,841 - 8,270,646	
TL	4	qTL4-1	10.2	3.2	0.65	0.56	98.8	97.5 - 102.9	178,383,156	177,375,782 - 180,773,788	
TL	5	qTL5-1	6.3	1.9	0.59	0.21	1.5	1.0 - 3.0	1,105,596	187,261 - 1,818,135	
TL	6	qTL6-1	6.5	2.0	-0.64	0.20	41.6	38.5 - 44.4	109,385,580	107,178,536 - 111,026,163	
TL	7	qTL7-1	12.6	3.9	-0.80	-0.05	56.3	53.1 - 59.5	106,655,101	40,303,561 - 115,968,999	Q28US-NAM
TL	7	qTL7-2	10.6	3.3	-0.73	0.00	145.0	143.5 - 145.6	174,321,800	173,803,732 - 174,506,396	
TL	8	qTL8-1	9.7	3.0	-0.77	-0.05	75.7	71.8 - 79.9	119,036,853	109,726,324 - 129,122,663	Q31US-NAM
TL	9	qTL9-1	30.7	10.1	1.33	0.30	85.3	80.2 - 87.2	139,410,209	133,933,227 - 140,341,356	
TL	10	qTL10-1	11.2	3.5	1.32	-0.43	39.5	38.5 - 41.0	98,999,149	87,097,012 - 109,133,400	
TPL	1	qTPL1-1	4.6	1.6	0.12	-0.59	26.6	25.0 - 34.5	11,944,272	10,902,621 - 15,737,577	NA
TPL	1	qTPL1-2	13.1	4.7	0.48	-0.12	151.9	148.6 - 153.5	281,524,945	279,679,264 - 283,191,861	NA
TPL	2	qTPL2-1	6.4	2.2	-0.34	0.07	9.0	5.9 - 11.8	2,619,855	2,082,275 - 3,388,685	NA
TPL	3	qTPL3-1	18.7	6.8	0.63	-0.21	113.6	112.6 - 114.2	205,261,445	204,461,392 - 205,409,189	NA
TPL	4	qTPL4-1	6.5	2.3	-0.28	-0.18	37.1	32.6 - 39.4	11,828,739	10,454,969 - 14,386,238	NA
TPL	5	qTPL5-1	14.8	5.3	0.37	0.35	82.8	77.8 - 87.0	137,930,872	78,307,703 - 152,431,048	NA
TPL	5	qTPL5-2	8.1	2.8	0.34	-0.12	166.5	161.7 - 172.2	214,949,620	213,268,517 - 217,710,870	NA
TPL	6	qTPL6-1	5.1	1.8	0.27	-0.12	2.2	1.0 - 7.9	3,045,425	271,281 - 7,046,668	NA
TPL	6	qTPL6-2	9.6	3.4	-0.37	0.06	81.0	79.2 - 82.7	154,897,834	153,785,475 - 155,626,565	NA
TPL	7	qTPL7-1	5.0	1.8	0.27	-0.16	32.1	26.5 - 36.2	10,516,953	8,173,317 - 14,367,769	NA
TPL	7	qTPL7-2	5.0	1.8	0.26	-0.17	123.1	118.5 - 128.5	167,909,787	165,385,654 - 170,496,503	NA
TPL	9	qTPL9-1	15.4	5.5	0.45	0.05	39.6	37.8 - 41.3	21,967,530	20,829,178 - 22,478,819	NA

[a]Positive and negative values indicate that the teosinte allele has a positive or negative effect on the corresponding trait

[b]genetic position range for the2-LOD support interval

[c]physical position range for the 2-LOD support interval

[d]Wu *et al*. (2016) performed joint linkage mapping for tassel length and tassel primary branch number in both CN NAM and US NAM, and the overlapped QTLs were listed. The other three traits, TBL, TPL and TBA, were not investigated in Wu *et al.*'s study. NA, not available.

Table S5 Phenotypic effect analyses of NILs at *ra1* locus on chromosome 7

Trait	Mean ± sd		Sample size		P-value
	NIL^{maize}	$NIL^{teosinte}$	NIL^{maize}	$NIL^{teosinte}$	
TBN	7.7 ± 3.00	9.9 ± 3.62	81	84	3.30E-05
TL (cm)	29.6 ± 2.44	27.9 ± 2.28	79	79	2.46E-05
TBL (cm)	19 ± 1.60	17.1 ± 1.57	78	79	3.35E-12
LL (cm)	72.5 ± 3.63	69.5 ± 3.83	64	62	2.12E-05
DTA	60.8 ± 2.08	59.5 ± 2.01	79	81	5.47E-05
TPL (cm)	5.2 ± 1.94	5.2 ± 1.66	77	79	0.94
LN	13.9 ± 0.94	13.6 ± 0.85	67	66	0.17
LW (cm)	8.3 ± 0.80	8.5 ± 0.80	76	76	0.14
PH (cm)	128.3 ± 10.68	127.5 ± 9.303	80	82	0.61
EH (cm)	42.7 ± 7.67	40.9 ± 7.40	77	79	0.14

TBN, tassel branch number; TL, tassel length；TBL, tassel branch length; LL, leaf length; DTA, days to anthesis; TPL, tassel peduncle length; LN, leaf number; LW, leaf width; PH, plant height; EH, ear height. Phenotype values are expressed as mean ± SD.

Table S6 Primers used for *bif2* sequencing

Region	F (5’-3’)	R (5’-3’)
A1	TGAACCACCGACCATTATCT	CATGATCACTGGCGTCAAAT
A2	GCCGTTTCACAGACACATTG	GGTTAGGCAGGGATTAAGCA
A3	TTGCCGTCCCCGACAAAGTTA	TCGCTTGCCGTGTCTCCGA
A4	ATTGACGGATCTGCCCCTCC	CGTATCTGTAGGCTGTAGCA
A5	GCGAAGAAGAAGCTGGAGCA	TGGCGAGTGATGCAACTAGG
A6	CGTGTTCCTGTACGAGCTCC	GCGCCATTAATGTAATCGCC
A7	CTGCTCGGTCGAGGTCTTCT	CAGGTTTAGGTCCGTTTGGA

New Phytologist

Table S7 Nucleotide diversity statistics at *bif2*

Region	Maize						Teosinte						$\pi^{maize}/\pi^{teosinte}$ (%)
	N	L	S	π	θ	Tajima's D	N	L	S	π	θ	Tajima's D	
A1	25	599	32	0.0063	0.0141	**-2.072**	8	552	68	0.0472	0.0457	-0.401	13.4**
A2	27	658	18	0.0042	0.0071	-1.445	9	634	37	0.0200	0.0215	-0.706	20.9**
A3	28	554	13	0.0067	0.0060	0.115	17	541	39	0.0181	0.0213	-0.720	37.2*
A4	12	594	27	0.0152	0.0151	0.045	10	548	46	0.0223	0.0297	-1.218	68.3
A5	27	683	13	0.0036	0.0049	-0.913	8	677	14	0.0060	0.0080	-1.516	59.9
A6	25	482	11	0.0058	0.0060	-0.138	13	482	19	0.0118	0.0127	-0.317	49.3
A7	20	680	38	0.0062	0.0158	**-2.402**	14	329	17	0.0164	0.0162	0.047	37.9

N, number of sequence; L, the length of a given locus; S, number of segregating sites; π, nucleotide diversity per site; θ, nucleotide polymorphism per site; statistically Tajima's D test values are in bold. *, $P< 0.05$; **, $P< 0.01$.

New Phytologist

Table S8 Molecular markers used for QTL effect validation and *qTL9-1* fine mapping

Marker	Locus	Type*	Chr	Pos (bp)	F (5'-3')	R (5'-3')
M1	*qTL9-1*	InDel	chr9	134,301,228	GTTTCACCTGTCTTAGCACCA	GGACAGAAGTTGAACCCAGTATC
M2	*qTL9-1*	InDel	chr9	136,176,830	CCACCCTTGGAAACAGGTAAT	GTATTTTCTAGTCGGATATCAG
M3	*qTL9-1*	InDel	chr9	136,701,239	CTGAGCTCCTGATTTCTTGCTCTC	AAACATTTAATCCAACAGCCCAGA
M4	*qTL9-1*	InDel	chr9	137,491,249	TCATCTTCCCCACAAATTTTCATT	GACTGACAACTCAGATTTCACCCA
M5	*qTL9-1*	InDel	chr9	137,765,651	GGCTACTTACGAATCTAAG	AGTCCACCTCCAACTTATAT
M6	*qTL9-1*	InDel	chr9	137,987,139	GCGCAACTTGAGTCCATGC	CTGCTAGCTGGTGGACGA
M7	*qTL9-1*	InDel	chr9	138,754,172	CTCACGCCGATGGATTTC	CTGCGGGTCCAGTTTAGTCT
M8	*qTL9-1*	InDel	chr9	139,663,969	GCCCTATTGTGGCATGCAAT	CGTGATTGGTCCAAAGTGAC
M9	*qTL9-1*	CAPs (MspI)	chr9	139,700,879	ACCAGCAGCACACTCATGG	AAAACAGGCTTGTTCGTTCCT
M10	*qTL9-1*	InDel	chr9	139,835,817	GAGTCGAATCCAGAACGTCT	CAGCGGTCCAGTTGCAGC
M11	*qTL9-1*	InDel	chr9	140,025,021	TTCAGGAGGATGGAGTACCTCT	TCACGGAACGAGACGTAAGA
M12	*qTL9-1*	InDel	chr9	140,213,709	GACGCAGCAAGTCAAAACAT	CTGTTAGAAATGCTCATCAGG
M13	*qTL9-1*	CAPs (BstNI)	chr9	140,344,017	CGTTGTCCATCATTGTTCTCG	GCTGAACATTATGTGGCACTG
TBN-1	*qTBN7-1*	InDel	chr7	105,911,296	ATCAGCATCCTCCATTCACACAT	CGGGAAATGCTAGAATTATGCTGA
TBN-2	*qTBN7-1*	InDel	chr7	112,028,371	ATTGGATGGATGTGCGCAGT	TCTCTGGCGTCGACATTGAT

* InDel represents insertion and deletion markers; restriction enzymes used for CAPs markers were listed in brackets.

Table S9 Functional annotation of the 21 candidate genes in the 513-kb target region of *qTL9-1*

Gene model	Gene start (bp)*	Gene end (bp)*	Annotation
GRMZM2G103270	139,698,112	139,701,345	ankyrin repeat family protein
GRMZM2G103247	139,705,351	139,707,757	protein phosphatase 2C
GRMZM2G063151	139,744,606	139,748,920	initiator-binding protein
GRMZM2G114126	139,761,157	139,765,745	oxysterol-binding protein
GRMZM2G098784	139,836,105	139,839,851	GRAS family transcription factor
GRMZM2G151992	139,844,221	139,846,531	Haloacid dehalogenase-like hydrolase (HAD) superfamily protein
GRMZM2G700034	139,854,927	139,855,509	unknown
GRMZM2G103595	139,929,569	139,930,729	ROTUNDIFOLIA like 8
GRMZM2G064710	139,942,775	139,945,257	unknown
AC202144.5_FG001	139,943,334	139,945,062	unknown
GRMZM2G492252	139,947,777	139,955,628	RING/U-box superfamily protein
GRMZM2G045084	139,961,781	139,963,488	zinc finger family protein
GRMZM2G045102	139,968,673	139,969,693	unknown
GRMZM5G888263	139,980,251	139,983,891	outer membrane protein, OMP85 family
GRMZM2G145313	140,014,798	140,016,394	avr9/Cf-9 rapidly elicited protein
GRMZM2G089803	140,023,280	140,026,450	ent-kaurenoic acid hydroxylase 2
GRMZM2G164160	140,111,984	140,120,409	calmodulin depedent protein kinases
GRMZM2G164136	140,123,391	140,127,499	myosin heavy chain-related
GRMZM2G053199	140,162,005	140,163,880	Ser/Thr protein phosphatase family protein
GRMZM2G700036	140,166,647	140,167,741	unknown
GRMZM2G151934	140,212,757	140,217,688	LIM domain-containing protein

* The physical positions of genes are according to maize B73 reference genome APGv2

References

Acosta IF, Laparra H, Romero SP, Schmelz E, Hamberg M, Mottinger JP, Moreno MA, Dellaporta SL. 2009. *tasselseed1* is a lipoxygenase affecting jasmonic acid signaling in sex determination of maize. *Science* **323**: 262-265.

Bai F, Reinheimer R, Durantini D, Kellogg EA, Schmidt RJ. 2012. TCP transcription factor, *BRANCH ANGLE DEFECTIVE 1* (*BAD1*), is required for normal tassel branch angle formation in maize. *Proceedings of the National Academy of Sciences, USA* **109**: 12225-12230.

Bolduc N, Yilmaz A, Mejia-Guerra MK, Morohashi K, O'Connor D, Grotewold E, Hake S. 2012. Unraveling the *KNOTTED1* regulatory network in maize meristems. *Genes & Development* **26**: 1685-1690.

Bomblies K, Wang R-L, Ambrose BA, Schmidt RJ, Meeley RB, Doebley J. 2003. Duplicate *FLORICAULA/LEAFY* homologs *zfl1* and *zfl2* control inflorescence architecture and flower patterning in maize. *Development* **130**: 2385-2395.

Bommert P, Nardmann J, Vollbrecht E, Running M, Jackson D, Hake S, Werr W. 2005b. *thick tassel dwarf1* encodes a putative maize ortholog of the Arabidopsis *CLAVATA1* leucine-rich repeat receptor-like kinase. *Development* **132**: 1235-1245.

Bortiri E, Chuck G, Vollbrecht E, Rocheford T, Martienssen R, Hake S. 2006. *ramosa2* encodes a LATERAL ORGAN BOUNDARY domain protein that determines the fate of stem cells in branch meristems of maize. *Plant Cell* **18**: 574-585.

Chatterjee M, Tabi Z, Galli M, Malcomber S, Buck A, Muszynski M, Gallavotti A. 2014. The boron efflux transporter *ROTTEN EAR* is required for maize inflorescence development and fertility. *Plant Cell* **26**: 2962-2977.

Chuck G, Meeley R, Hake S. 2008. Floral meristem initiation and meristem cell fate are regulated by the maize AP2 genes *ids1* and *sid1*. *Development* **135**: 3013-3019.

Chuck G, Meeley R, Irish E, Sakai H, Hake S. 2007. The maize *tasselseed4* microRNA controls sex determination and meristem cell fate by targeting *Tasselseed6/indeterminate spikelet1*. *Nature Genetics* **39**: 1517-1521.

Chuck G, Meeley RB, Hake S. 1998. The control of maize spikelet meristem fate by the APETALA2-like gene *indeterminate spikelet1*. *Genes & Development* **12**: 1145-1154.

Chuck G, Muszynski M, Kellogg E, Hake S, Schmidt RJ. 2002. The control of spikelet meristem identity by the *branched silkless1* gene in maize. *Science* **298**: 1238-1241.

Chuck G, Whipple C, Jackson D, Hake S. 2010. The maize SBP-box transcription factor encoded by *tasselsheath4* regulates bract development and the establishment of meristem boundaries. *Development* **137**: 1243-1250.

Chuck GS, Brown PJ, Meeley R, Hake S. 2014. Maize SBP-box transcription factors *unbranched2* and *unbranched3* affect yield traits by regulating the rate of lateral primordia initiation. *Proceedings of the National Academy of Sciences, USA* **111**: 18775-18780.

DeLong A, Calderon-Urrea A, Dellaporta SL. 1993. Sex determination gene *TASSELSEED2* of maize encodes a

short-chain alcohol dehydrogenase required for stage-specific floral organ abortion. *Cell* **74**: 757-768.

Gallavotti A, Malcomber S, Gaines C, Stanfield S, Whipple C, Kellogg E, Schmidt RJ. 2011. *BARREN STALK FASTIGIATE1* is an AT-hook protein required for the formation of maize ears. *Plant Cell* **23**: 1756-1771.

Gallavotti A, Zhao Q, Kyozuka J, Meeley RB, Ritter MK, Doebley JF, Pè ME, Schmidt RJ. 2004. The role of *barren stalk1* in the architecture of maize. *Nature* **432**: 630-635.

Galli M, Liu Q, Moss BL, Malcomber S, Li W, Gaines C, Federici S, Roshkovan J, Meeley R, Nemhauser JL. 2015. Auxin signaling modules regulate maize inflorescence architecture. *Proceedings of the National Academy of Sciences, USA* **112**: 13372-13377.

Han J-J, Jackson D, Martienssen R. 2012. Pod corn is caused by rearrangement at the *Tunicate1* locus. *Plant Cell* **24**: 2733-2744.

Hartwig T, Chuck GS, Fujioka S, Klempien A, Weizbauer R, Potluri DPV, Choe S, Johal GS, Schulz B. 2011. Brassinosteroid control of sex determination in maize. *Proceedings of the National Academy of Sciences, USA* **108**: 19814-19819.

Je BI, Gruel J, Lee YK, Bommert P, Arevalo ED, Eveland AL, Wu Q, Goldshmidt A, Meeley R, Bartlett M. 2016. Signaling from maize organ primordia via *FASCIATED EAR3* regulates stem cell proliferation and yield traits. *Nature Genetics* **48**: 785-791.

Leonard A, Holloway B, Guo M. 2014. *tassel-less1* encodes a boron channel protein required for inflorescence development in maize. *Plant and Cell Physiology* **55**: 1044-1054.

McSteen P, Hake S. 2001. *barren inflorescence2* regulates axillary meristem development in the maize inflorescence. *Development* **128**: 2881-2891.

Pautler M, Eveland AL, LaRue T, Yang F, Weeks R, Je BI, Meeley R, Komatsu M, Vollbrecht E, Sakai H. 2015. *FASCIATED EAR4* encodes a bZIP transcription factor that regulates shoot meristem size in maize. *Plant Cell* **27**: 104-120.

Satoh-Nagasawa N, Nagasawa N, Malcomber S, Sakai H, Jackson D. 2006. A trehalose metabolic enzyme controls inflorescence architecture in maize. *Nature* **441**: 227-230.

Taguchi-Shiobara F, Yuan Z, Hake S, Jackson D. 2001. The *fasciated ear2* gene encodes a leucine-rich repeat receptor-like protein that regulates shoot meristem proliferation in maize. *Genes & Development* **15**: 2755-2766.

Thompson BE, Basham C, Hammond R, Ding Q, Kakrana A, Lee T-F, Simon SA, Meeley R, Meyers BC, Hake S. 2014. The *dicer-like1* Homolog *fuzzy tasse*l is required for the regulation of meristem determinacy in the inflorescence and vegetative growth in maize. *Plant Cell* **26**: 4702-4717.

Vollbrecht E, Springer PS, Goh L, Buckler IV ES, Martienssen R. 2005. Architecture of floral branch systems in maize and related grasses. *Nature* **436**: 1119-1126.

Whipple CJ, Hall DH, DeBlasio S, Taguchi-Shiobara F, Schmidt RJ, Jackson DP. 2010. A conserved mechanism of bract suppression in the grass family. *Plant Cell* **22**: 565-578.

Wu X, Li Y, Shi Y, Song Y, Zhang D, Li C, Buckler ES, Li Y, Zhang Z, Wang T. 2016. Joint−linkage mapping and GWAS reveal extensive genetic loci that regulate male inflorescence size in maize. *Plant Biotechnology Journal* **14**: 1551-1562.
